# Predicting Spatial and Temporal Gene Expression Using an Integrative Model of Transcription Factor Occupancy and Chromatin State

**DOI:** 10.1371/journal.pcbi.1002798

**Published:** 2012-12-06

**Authors:** Bartek Wilczynski, Ya-Hsin Liu, Zhen Xuan Yeo, Eileen E. M. Furlong

**Affiliations:** 1 Genome Biology Unit, European Molecular Biology Laboratory, Heidelberg, Germany; 2 Institute of Informatics, University of Warsaw, Warsaw, Poland; Weizmann Institute of Science, Israel

## Abstract

Precise patterns of spatial and temporal gene expression are central to metazoan complexity and act as a driving force for embryonic development. While there has been substantial progress in dissecting and predicting *cis*-regulatory activity, our understanding of how information from multiple enhancer elements converge to regulate a gene's expression remains elusive. This is in large part due to the number of different biological processes involved in mediating regulation as well as limited availability of experimental measurements for many of them. Here, we used a Bayesian approach to model diverse experimental regulatory data, leading to accurate predictions of both spatial and temporal aspects of gene expression. We integrated whole-embryo information on transcription factor recruitment to multiple *cis*-regulatory modules, insulator binding and histone modification status in the vicinity of individual gene loci, at a genome-wide scale during *Drosophila* development. The model uses Bayesian networks to represent the relation between transcription factor occupancy and enhancer activity in specific tissues and stages. All parameters are optimized in an Expectation Maximization procedure providing a model capable of predicting tissue- and stage-specific activity of new, previously unassayed genes. Performing the optimization with subsets of input data demonstrated that neither enhancer occupancy nor chromatin state alone can explain all gene expression patterns, but taken together allow for accurate predictions of spatio-temporal activity. Model predictions were validated using the expression patterns of more than 600 genes recently made available by the BDGP consortium, demonstrating an average 15-fold enrichment of genes expressed in the predicted tissue over a naïve model. We further validated the model by experimentally testing the expression of 20 predicted target genes of unknown expression, resulting in an accuracy of 95% for temporal predictions and 50% for spatial. While this is, to our knowledge, the first genome-wide approach to predict tissue-specific gene expression in metazoan development, our results suggest that integrative models of this type will become more prevalent in the future.

## Author Summary

Development is a complex process in which a single cell gives rise to a multi-cellular organism comprised of diverse cell types and well-organized tissues. This transformation requires tightly coordinated expression, both spatially and temporally, of hundreds to thousands of genes specific to any given tissue. To orchestrate these patterns, gene expression is regulated at multiple steps, from TF binding to *cis*-regulatory modules, general transcription factor and RNA polymerase II recruitment to promoters, chromatin remodeling, and three-dimensional looping interactions. Despite this level of complexity, the regulation of gene expression is typically modeled in the context of transcription factor binding and a single enhancer's activity as this is where the majority of experimental data is available. Recent advances in the measurement of chromatin modifications and insulator binding during embryogenesis provide new datasets that can be used for modeling gene expression. Here we use a Bayesian approach to integrate all three levels of information to combine the activity of multiple regulatory elements into a single model of a gene's expression, implementing an expectation maximization strategy to overcome the problem of missing data. Importantly, while the data for histone modifications and insulator binding represents merged signals from all cells in the embryo, the model can extract cell type specific and stage-specific predictions on gene expression for hundreds of genes of unknown expression.

## Introduction

**Figure 1:**
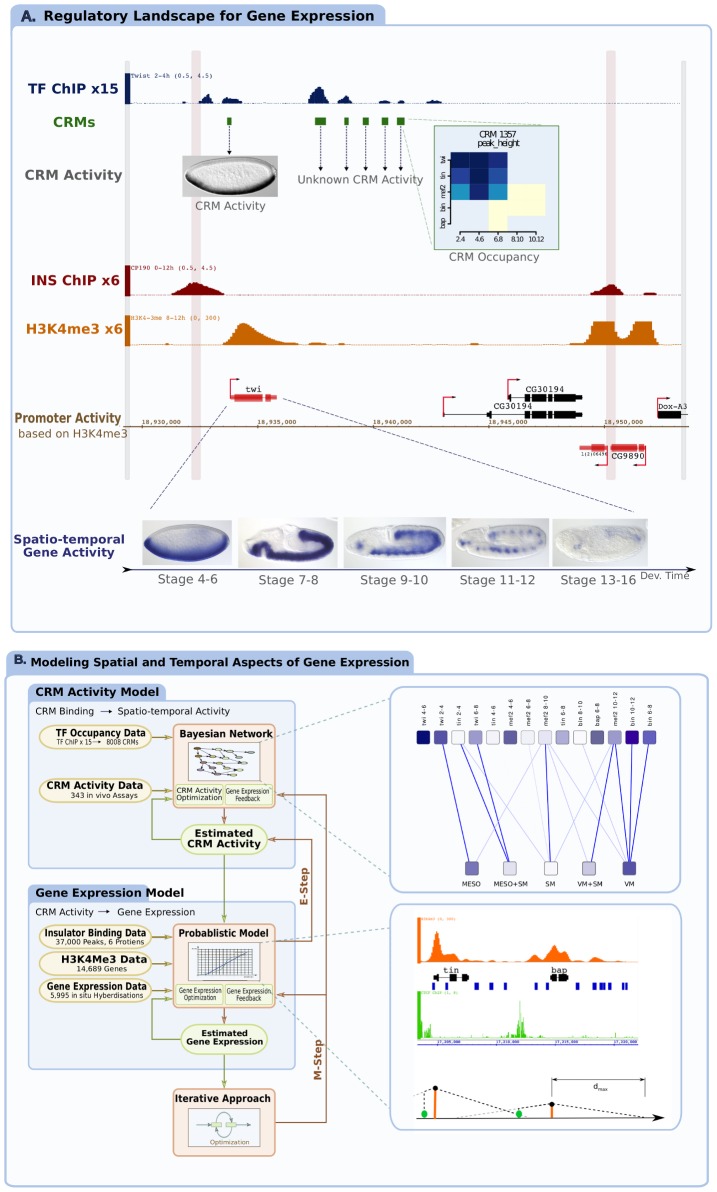
Generating a predictive model of spatio-temporal gene expression. (**a**) A typical genomic locus within the *Drosophila* genome. Depicted tracks represent, from top to bottom: Transcription factor (TF) binding (log2 ChIP-chip signal shown for one factor, blue), computed *cis*-regulatory modules (CRMs) from 15 developmental conditions (green). A zoomed heat map shows a detailed view of TF binding for one CRM for all 5 TFs and 5 time-points, the level of blue represents the degree of ChIP enrichment in log2. Insulator (INS) binding is shown in red (ChIP signal shown for CP190, one of 6 factors in dark red), Histone H3K4me3 for a selected time-point (orange) and gene models from RefSeq are indicated in black (inactive genes) or red (active genes) depending on the level of H3K4me3 signal. The boundaries of insulator occupancy places all CRMs in the vicinity of three genes, *twi* and *CG30194* and *l(2)06496*, while the enriched H3K4me3 signal at the *twi* and *l(2)06496* promoter indicates that they are the only genes actively expressed genes at these stages. The activity of only one enhancer is known within this locus (*twi*-PE). The spatio-temporal expression patterns of the *twi* gene is shown, characterized by in-situ hybridization. (**b**) A schematic representation of the iterative Bayesian modeling approach. The model consists of two major components joined through iteration of the EM algorithm: A Bayesian network that uses TF occupancy data (ChIP) and TF activity data (from transgenic reporter assays) to model CRM activity (an exemplary network topology that was a result of an optimization run is shown in a separate panel); a probabilistic model that uses insulator occupancy, promoter activity, CRM occupancy and estimates of CRM activity to model spatio-temporal gene expression. Separate panel includes all data used for an exemplary locus containing Tinman and Bagpipe genes. It is an interesting case as both genes are expressed in different times and sub-tissues originating from the mesoderm. In essence, the model estimates the probability of a gene's activity as a function of all data between the two insulator elements (green Chip signal in the inlay panel). An expectation maximization step (EM) is used to iteratively improve both the BN topology, CRM activity predictions, maximum CRM-gene distance (dmax), and the gene expression predictions until a local maximum of the likelihood is reached.

Gene expression is regulated through the interplay of transcription factors binding to *cis*-regulatory modules (CRMs), chromatin modifications and the basal transcriptional machinery recruited to promoter elements. CRMs function as discrete regulatory elements [1], [2], that can act at varying genomic distances from their target genes [3]. Despite recent advances in our understanding of the regulatory steps of transcription, the ability to predict both spatial and temporal aspects of gene expression remains surprisingly limited. Efforts in this direction can be broadly divided into two groups: (1) Predicting *cis*-regulatory or enhancer activity, where recent studies in yeast [4] *Drosophila* [5]–[7] and *C.elegans* [4] have made substantial progress. In one such study the tissue specificity of the neighboring gene's expression was used to guide the search for specific TF combinations [7], while in another the combination of sequence motif matches was used to predict gene expression [4]. Although, these are important steps, integrating the activity of multiple *cis*-regulatory elements that regulate overlapping or distinct aspects of a gene's spatio-temporal expression remains a key challenge (Fig. 1a, Fig. S1). (2) Using chromatin state dynamics to predict gene expression [8]–[12]), with or without information on transcription factor (TF) and insulator data. For example, in *Drosophila* a logistic regression was used to predict temporal (not tissue-specific) gene expression in embryogenesis [11], showing a performance better than random for 23.6% genes, with a 2.5 fold enrichment over control experiments where the connectivity between TFs and their targets was reshuffled. In *c.elegans* an SVM classifier was used for a similar task of discerning highly and lowly expressed transcripts based on measured chromatin marks [13], although tissue specificity was not examined. This approach, based on transcripts and chromatin marks in their immediate vicinity (+/−4 kb) achieves high accuracy (average AUC for all stages = 0.82), reflecting the strong correlation between transcription and chromatin marks on the gene body, such as the H3K79 methylation and Pol II occupancy consistent with the results by Karlic *et al* [14]. However, while virtually all regulatory elements appear to reside within 5 kb of the transcriptional start site (TSS) in *C. elegans*, this is not the case in other species.

In *Drosophila*, mouse and humans there are many examples of remote CRMs acting at large distances from the TSS [15]–[18] spanning many intervening genes [19], [20], where large chromatin loops are thought to bring the enhancers and the target gene's promoter in close physical proximity [21]. In addition, genes, especially developmental regulators, are controlled by multiple CRMs, giving rise to partially overlapping patterns of activity [22], [23]. In order to capture, and thereby predict, the full spectrum of a gene's spatial expression, two key issues need to be addressed directly: (1) accurately linking CRMs to their target genes and (2) integrating the activity from multiple CRM, as is done naturally for most developmental genes in multicellular species. There is currently very little biological information or understanding of how the activity of multiple elements is integrated at the promoter level. While some studies have suggested that each CRM acts in an additive manner so that the gene's expression pattern is the simply sum of all elements, other studies have shown that the gene can be expressed in a broader [8], [24]) or more restricted [9], [10] spatial domain than the sum of its individual regulatory elements. It is therefore currently not clear how best to integrate separate computational models of *cis*-regulatory elements to accurately reflect this convergence of regulatory information controlling a gene's expression *in vivo*. These difficulties have limited spatial predictions of gene expression to a small number of very well characterized genes [6], [25], or more globally to focusing on predicting on-off states in single cell systems [26], [27], thereby circumventing the inherent complexity of spatial expression within a multicellular organism.

High-resolution ChIP-chip or ChIP-seq approaches facilitate the mapping of distant regulatory elements based on transcription factor occupancy [26]–[32], co-factor binding [33] or chromatin marks [34], providing new possibilities to develop better predictive models of global gene expression patterns. However, there are still several levels of information missing, including a complete catalog of all enhancers active during specific stages of development, information on the identity and timing of the TFs recruited to each enhancer, cell-type specific information on chromatin status, the activity state of the associated target gene and a general lack of information on the physical association of CRMs with promoter elements. Despite this incomplete knowledge, we asked if the current level of information is sufficient to accurately predict spatio-temporal gene expression within the context of a multicellular embryo, reasoning that the predictive power of the model should only improve as more information becomes available.

## Results

We developed a probabilistic model, integrating diverse types of data generated from whole embryos and thereby containing mixed signals from many tissues, to predict both spatial and temporal aspects of gene expression, with particular emphasis on the mesoderm and derived muscle types. More specifically, using *Drosophila* embryogenesis as a model system, we integrated six types of data relevant to transcriptional regulation: (i) 19,000 TF binding peaks derived from ChIP-chip experiments for mesoderm specific TFs, clustered into 8008 non-overlapping *cis*-regulatory modules (ChIP-CRMs), (ii) spatio-temporal activity data for 343 CRMs from *in vivo* transgenic reporter assays, (iii) the genomic distance of CRMs in relation to transcriptional start sites, (iv) 37,923 occupancy peaks for 6 insulator binding proteins, (v) H3K4me3 enrichment measured for promoter regions of 14689 genes, and (vi) spatio-temporal expression of 5,995 genes derived from *in-situ* hybridization (see Table S1 for a detailed data description). Note, as chromatin modifying enzymes for canonical histones and insulator binding proteins are ubiquitously expressed, the whole embryo data from (iv) and (v) does not contain any inherent cell-type specific (spatial) information, and (v) represents merged temporal signal over the entire period of embryogenesis, which is 24 hr in *Drosophila*. TF occupancy (i) and gene expression (vi) data provide information on potential regulatory input and the final spatio-temporal output, respectively, but little means to connect the two, highlighting the need to integrate diverse layers of information.

Previous studies suggest that *cis* regulatory elements function, to a large extent, independently of each other [35]. Assuming that this is correct, there are two natural levels to model gene expression based on: (i) the relationship between TF occupancy and CRM activity and (ii) the relationship between models of multiple CRMs' activity and a gene's expression (Fig. 1a). This first step was recently addressed using support vector machine (SVM) models, which demonstrated that TF occupancy alone is sufficient to predict spatio-temporal CRM activity during mesoderm development [5]. It was postulated [36] that the same method could in principle be adapted to model gene expression prediction, although this would require linking CRMs to their appropriate target genes and integrating inputs from multiple CRM models to reflect a target gene's expression. Taking advantage of the wealth of data on TF occupancy at mesodermal CRMs [5], we tested this assumption by building a simple additive model that assigns each CRM to the nearest gene and then sums the SVM prediction scores for all assigned CRMs to obtain a spatio-temporal expression prediction at the gene level. Overall, the predictions were of poor quality (Fig. S2), indicating that a model based on these simple assumptions does not reflect the biological complexity of the system. Using well-characterized gene loci to examine why the model failed revealed that enhancers do not always regulate the nearest gene, but often a more distant gene (Fig. 1a *twist* locus) or can even act across an intervening inactive gene to reach its appropriate target (Fig. S1a *bagpipe* locus). Such inactive ‘bystander’ genes [37] can be located within the intron of a target gene (Fig. S1c *Fas3* locus) or vice versa ( *CG6981*), further confounding the problem of appropriate target gene assignment. This demonstrates the need to move to a more integrative model that includes information on promoter activity (H3K4me3 enrichment) and insulator occupancy within a gene locus. As insulator binding proteins mediate long-range regulatory interactions between enhancers and their target genes [3], [38], we reasoned that insulator occupancy could improve the ability to recognize ‘bystander genes’, while the presence of H3K4me3 at promoters will identify active genes within the vicinity of active CRMs.

To deal with this complexity, we applied a Bayesian model to probabilistically integrate diverse types of data in an iterative manner, which has the advantage of being able to cope with uncertainty and incompleteness within each dataset using conditional probabilities. The model consists of three components (Fig. 1b): (i) a Bayesian Network (BN) that describes the probability of a CRM being active in a tissue or time-point as a function of its occupancy by different TFs, (ii) a custom probabilistic model that describes the probability of a gene being expressed at a given stage and tissue depending on the activity of surrounding CRMs, the location of CRMs and insulators relative to the promoter, and the activity state of the promoter (Fig. 1), and (iii) an expectation maximization (EM) procedure [39] that functions to find an optimal set of parameters within the overall Bayesian model, iterating between the BN and custom model until convergence. To accurately predict gene expression, the model must be able to cope with dynamic changes in the regulatory context of genes, which determines their activity state at different stages of development and in different tissues. To account for this, we trained the model using spatio-temporal expression information of 5,082 non-ubiquitous genes generated from large-scale *in-situ* hybridization experiments [40], describing when and where genes are expressed during embryogenesis. As a proof-of-principle we focused on five temporal windows of development and five tissue classes (10 prediction classes; Supplementary text S1.).

**Figure 2:**
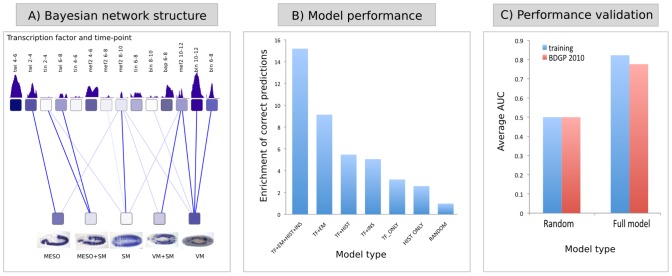
An iterative Bayesian model can accurately predict gene expression. (**a**) The learned Bayesian network topology reveals regulatory relationships between transcription factors (TFs) and specific tissues. Each node in the network represents TF occupancy data (TF-f and time-T) or a specific activity class (tissue or time-period). The edges represent the probability of a CRM being active as a function of a particular binding event, with darker blue lines having the highest probability. Predicted activity in Meso class is dependent on Twist (Twi) binding to a CRM at 2–4 hr, while VM activity depends on Biniou (Bin) occupancy at two time-points. Meso = unspecified mesoderm, VM = visceral muscle, SM = somatic muscle. (**b**) Histogram showing average enrichment of correct predictions within the top 2% of genes with highest posterior probability from all 10 activity classes, where a 15-fold enrichment is obtained using the iterative trained model including all datasets. This enrichment steadily decreases as one or more datasets are removed, going form a 9-fold enrichment when omitting insulator binding and H3K4me3 activity data (TF+EM), ∼6-fold enrichment when TF binding is used with either insulator or H3K4me3 data without the iterative EM procedure, to an ∼3-fold enrichment when TF binding data or histone marks alone are used. (**c**) Validation of the cross-validated model using *in-situ* hybridzation data for 600 genes not included in the training set. The average area under the curve (AUC) for all 10 classes ranges from 0.82 (training) to 0.78 (new data).

In more detail, the first component, modeling CRM activity as a function of TF binding events, was achieved using a BN, allowing for accurate representation of conditional probability (Fig. 2a, described in detail in Supplementary text S1 – in “Layer 1-TF binding” and “Layer 2-CRM activity”). The model uses measured TF binding events on CRMs as input (from ChIP-chip data) and spatio-temporal CRM activity data as output (from *in vivo* transgenic-reporter assays) (depicted in Fig. 1a). The nodes within the BN are of two types: specific TF binding events (factor-F at time-point-T, representing 15 variables) and activity classes (tissue or time-point, representing 10 variables). Each edge between nodes represents the probability of a CRM being active in a given activity class as a function of a particular binding event (e.g. CRM activity in tissue-A depends on the binding of factor-F at time-point-T). The correct topology of connections was reconstructed using the Bayesian Dirichlet equivalence score as implemented in the BNfinder software [41]. Once the most likely topology was known, the conditional probabilities of CRM activity in different classes (temporal and spatial) were calculated from the training data using the maximum likelihood principle. The trained BN and the conditional probability distributions were then used to provide probability estimates for the spatio-temporal activity of all 8008 CRMs, not only the 147 used in the training dataset. Based on these probability estimates, we compared the BN model with the previously published SVM approach [5]. Overall, our model gives slightly better predictions of previously unseen CRM activity (Fig. S3b), even though it was not explicitly optimizing the accuracy at the CRM level. In addition, unlike ‘black box’ type models such as SVMs, the learned BN network topology provides interpretable insights into the most important TF binding events for each spatio-temporal activity. For example, the BN revealed that Biniou (a FoxF TF) enhancer occupancy is the key predictive signal for visceral muscle activity (Fig. 2a), which matches the known essential role of this TFs for visceral muscle development from genetic studies [2].

The second component of the Bayesian model addresses how genes integrate probabilistic signals from one or many CRMs by relating this information to known gene expression patterns within the training set (described in detail in Supplementary text S1 – “Layer 3-gene activity”). For each gene, we consider the location of its transcriptional start site (TSS) and the CRMs present in its broad environment (+/−100 kbp, where there is one gene per ∼8 kb in the *Drosophila* genome). As the majority of known *Drosophila* enhancers are located within +/−20 kb of their target gene's promoter, the probability of activation decreases linearly with respect to the distance from the TSS. The only parameter that the model fits is the maximal distance between a CRM and the TSS within a +/−100 kbp window. To facilitate linking CRMs to their appropriate target gene, the model integrates information on the occupancy of six insulator binding proteins [42] relative to the location of CRMs and surrounding genes (Fig. 1a). As insulator proteins can block enhancers from inappropriately activating nearby promoters [43], CRM-promoter interactions are considered blocked if they operate across an insulator boundary (see Methods). To obtain a probability for a promoter being in an active or inactive state, we used the presence of H3K4-trimethylation ChIP-seq signal at promoters as an indicator of promoter activity [44] (Fig. 1a, Fig. S1, methods). The model requires both an active promoter and at least one active CRM to activate a gene in a given spatio-temporal context. The classifier accuracy was determined using the area under a receiver-operator curve (AUC) for varying posterior probabilities of gene activation. To train the model for tissue specific or developmental stage specific chromatin context, we used *in-situ* hybridization data of 5082 genes [40] to identify genes in specific spatio-temporal classes. For simplicity, expression patterns were divided into a number of binary classes: focusing on 5 tissue classes (mesoderm, somatic muscle, visceral muscle, mesoderm+somatic muscle and visceral+somatic muscle) and 5 time-windows (stages 4–6, 7–8, 9–10, 11–12, 13–16, spanning ∼85% of embryonic development). Separate variables were incorporated for a gene or CRM activity in each class, allowing each class to be evaluated individually using the probability of a gene to be expressed in a particular spatial or temporal domain.

The coupling between the two mentioned components of the model is through the intermediate layer representing the activity of the CRMs (Supplementary text S1 “Integrating the different layers of the model using iterative optimization”). Since the activity of the vast majority of the ChIP-defined CRMs is unknown, the variables in the intermediate layer are latent. Under this setting, an iterative Expectation maximization (EM) [39] procedure was used to facilitate using data of varying degrees of completeness at different levels of the model. The TF binding data is very extensive for all 8008 CRMs (at least within the scope of the five TFs), as is the insulator occupancy and promoter activity data, although the later two represent merged signals from mixed tissue types and have very low temporal resolution. Spatio-temporal expression data is available for a substantial number of genes (∼33% of predicted *Drosophila* genes), which contrasts with the scarcity of knowledge on CRM activity, which is available for only ∼4% of CRMs. This level of CRM activity data is sufficient to train a predictive model of CRM activity, using a BN (Fig. 2a) or SVM [5] approach. However, there is not a single gene in the *Drosophila* genome where the activity of all ChIP-defined CRMs in its vicinity are known. As such, there are no complete examples that could be used to fit a model representing convergence of multiple CRM activities to a single gene's expression. To address this, the activity of CRMs was consistently treated as a hidden variable in the model, and the CRM activity information was only used for model initialization. EM was used to iteratively improve both the CRM activity predictions and gene expression predictions (see methods and Fig. 1b), resulting in an effective model with local maximal likelihood.

By performing the EM procedure in a 10-fold cross-validation framework, we assessed the ability of the model to predict gene expression for genes not used for training. The average AUC value for all 10 prediction classes exceeds 0.8 (Fig. S4), a significant improvement over the simple additive SVM method (p-value<10^−7^; Fig. S3a). Importantly, the cross-validation estimated performance is comparable to that of the model trained on the full dataset (Fig. S5), indicating that the model is not over-fitted. The difference in AUC slightly underestimates the improvement of the model as it is based on predictions made for all genes, while only a minority of *Drosophila* genes are expected to be specifically expressed in each activity class and the majority of genes are correctly predicted not to be regulated by mesodermal CRMs. For example, from all 5082 *Drosophila* genes with characterized non-ubiquitous expression, only 137 have annotated expression in the activity class somatic muscle, 135 in mesoderm and 60 in VM [40]. Extrapolating these numbers to the entire genome estimates that the percentage of genes expressed in each activity class is in the range of 1–2%, excluding ubiquitously expressed genes. With this in mind, we examined the top 2% of predictions from the trained Bayesian model, which identified on average a 15-fold enrichment in gene expression in the predicted tissue compared to a random classifier, for all activity classes, with the best class having a 45-fold enrichments (Fig. 2b).

To investigate the most important aspects of the model's predictions, we compared the results to simpler approaches that do not use either chromatin state (insulator binding data or H3K4me3) or an EM procedure, all of which obtained inferior results (Fig. 2b, Fig. S6). Adding H3K4me3 promoter activity signal to TF binding, for example, reduces the number of false-positive predictions by 1.5 fold, thereby increasing the enrichment of correct predictions (Fig. 2b). The method also demonstrates improved performance over a simpler two-layer model predicting gene expression directly from ChIP peaks, skipping the intermediate CRM layer [45] (, Methods). Although this 2-layer model is not accurate enough to make reliable predictions, the approach can be very valuable for initiation of the EM algorithm in cases where there is no CRM activity database available. In many organisms obtaining information on CRM activity for a large number of regulator elements is difficult. We therefore tested whether our approach could provide comparable results without providing the measured activity of selected CRMs. To avoid random fluctuations we have used the gene expression data for genes with very closely (<500 bp) associated CRMs as a proxy for enhancer activity. While this is certainly introducing some erroneous information by both erroneous target assignment and by assigning total gene activity to only one selected enhancer, it seems to give only slightly worse results for classes with multiple genes associated to it (VM, SM, MESO, see Fig. S10).

**Figure 3:**
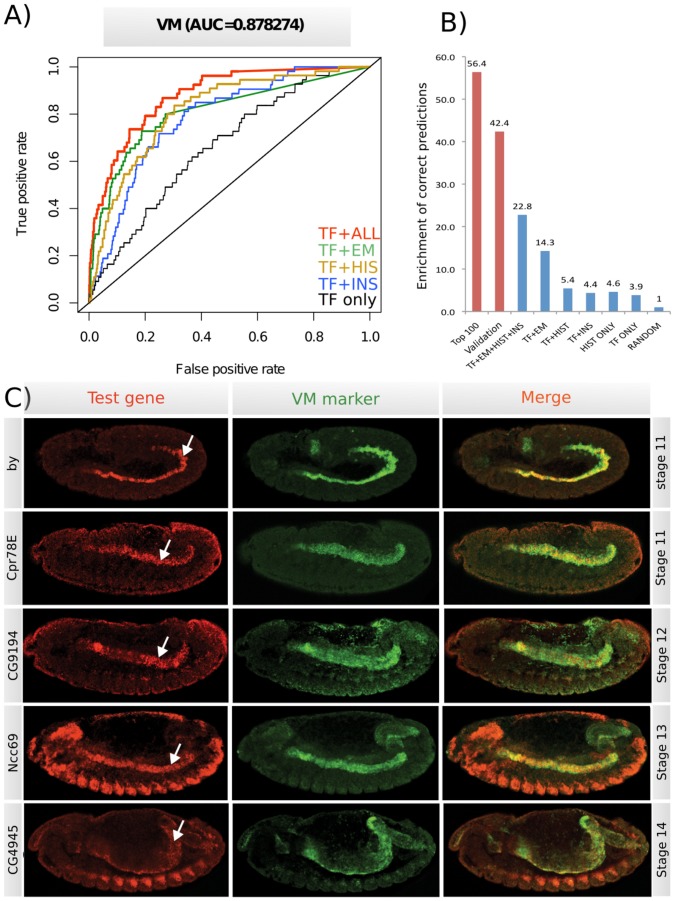
Validating spatio-temporal expression predictions in the visceral muscle. (**a**) Receiver Operator Curves (ROC) for the activity class visceral muscle (VM). The area under the curve (AUC) is 0.87 for the full iterative model using all data (TF+ALL), which becomes progressively lower for simpler models that either do not include chromatin data (TF+EM), or do not include the EM step (TF+His, TF+Ins). (**b**) Enrichment of correct predictions in the top (2%) of genes for different models and validation data. Blue bars present performance of different models using the training data for the visceral muscle activity class (VM). Red bars show analagous enrichment for the *in-situ* validated examples as well as for the top 100 predictions of genes expressed in VM, which were manually annotated based on the literature. (**c**) Embryo images showing double fluorescent *in-situ* hybridization against the gene with predicted expression (red) and a specific marker for VM (green, *biniou*), where overlapping gene expression in VM is shown in the merge panel. The white arrow points to the VM. All embryos are orientation with anterior to the left and dorsal up. *In-situ* data for all 22 genes tested are shown in Fig. S9.

To validate the true performance of the model we took advantage of spatio-temporal expression data for more than 600 genes not included in our training set that was part of the third release of the Berkeley *Drosophila* Genome Project (BDGP) *in-situ* database [40]. We used models trained on the whole training dataset and assessed their performance on the genes present only in the new dataset by calculating the AUC for each activity class (Fig. S8). The performance was comparable to the cross-validated-based estimates, with the average AUC of 0.78 (compared to 0.82; Fig. 2c). To further validate the quality of the trained model, we chose a tissue with a relatively restricted spatial expression, the visceral muscle (Fig. 3a, AUC 0.87), and manually curated the top 100 genes predicted to be expressed in this tissue (VM). Examining the literature and BDGP, we identified spatio-temporal expression for 46 of the 100 genes, 67% of which are expressed in visceral muscle, while the expression of the remaining 33% did not fit with our prediction (Dataset S8). We randomly selected 22 genes for which there was either no expression data available, or were apparent prediction errors from the model (within the 33%). Double fluorescent *in-situ* hybridization using a visceral muscle specific marker revealed that the timing of expression of 21 out of 22 genes match their temporal prediction (95%), while the expression of 50% match their spatial prediction (Fig. 3c, Fig. S9), representing a 42-fold enrichment in gene expression in visceral muscle compared to the 1.2% of genes annotated in the BDGP database (Fig. 3b). The high success rate of the model, despite the presence of inaccurate expression annotations within the training dataset, demonstrates the general robustness of this iterative approach.

## Discussion

This study represents a first attempt to build an integrative probabilistic genome-wide model that predicts both the spatial and temporal aspects of gene expression, within the context of a multicellular embryo. The Bayesian model integrates diverse types of genomics data, including transcription factor occupancy, chromatin modification and insulator binding information, using *in vivo* CRM activity information and gene expression data to train the model. In addition to predicting gene expression, introspection of the model's parameters reveals a number of additional insights. First, the iterative trained Bayesian network improved the accuracy of the previously published SVM approach for CRM and gene activity prediction [5] (Fig. S3), and recovered, without any prior information, the known dependencies between specific TFs and respective tissues. Second, through expectation-maximization, the model learns the optimal distance of a CRM to its target gene. This revealed extensive long-range enhancer activity, which may be much more widespread in *Drosophila* than previously anticipated. Although there are a handful of known enhancers acting >30 kb from their target gene [19], [20], the majority of CRMs identified to date are <+/−20 Kb of their target gene. This apparent close proximity, however, most likely reflects the current biases in how CRMs are identified in single gene studies, starting from the gene moving out, or in global studies where CRMs are typically linked to the closest proximal gene. The iterative Bayesian model revealed that CRMs as far as 50 kbp from the transcriptional start site have important contributions to accurately predict a gene's activity. Third, the model suggests that enhancer sharing between genes may be an inherent property of developmental enhancers where a CRM contributes to the predictions of 3 genes on-average. This observation, which came directly from the trained model, has recently been observed using an experimental technique to link CRMs to genes (chromatin conformation capture) [12], and has exciting implications for how transcriptional networks are regulated during development.

Taken together, this approach provides a method to move from descriptive ‘omics’ type data to predictive models of gene expression. Given the exponential increase in measurements of chromatin state and TF occupancy in the coming years, we expect this type of iterative analyses to become increasingly useful as a complement to ongoing attempts to map global gene expression patterns by experimental approaches and as a tool to uncover novel properties of transcriptional regulation.

## Methods

### Data material

CRM occupancy data and CAD database were used as published by Zinzen et al. [5]. Gene expression patterns were obtained from the BDGP *in-situ* hybridization database [46] - release 2 served as the training data, while release 3 (beta release downloaded on May 27th 2010) was used as the testing dataset. Only genes with tissue specific expression (excluding ubiquitous and maternal expression) were analyzed. Anatomical terms from BDGP were grouped into more general classes (mesoderm, somatic muscle, visceral mesoderm), similarly to the procedure used by Zinzen et al. [5]. Temporal classes were based on the staged groups used by the BDGP in their annotations (st.4–6, 7–8, 9–10,11–12,13–16). Whole embryo ChIP-seq data of histone H3K4 tri-methylation was from ModEncode [47] for three time-points: 4–8 h, 8–12 h and 12–16 h (ModEncode sample IDs:790, 791, 792). Averaged processed signal was calculated for a region surrounding all transcriptional start sites (−100,+400 bp from TSS) and then discretized into low and high values (threshold 0.3) for training the Bayesian network. Whole embryo ChIP-chip data for the six insulator proteins was obtained from Negre et al. [42] (using a 1% FDR).

### The model structure

The Bayesian model is composed of three main layers of different nature. The first layer represents variables corresponding to Transcription Factor binding to CRMs; second represents the CRMs activity under different conditions and the third is concerned with gene activity under the same set of conditions. We made the assumption that the only True causal connections are either coming from the first to second layer (TF binding causing CRM activity) or from the second to third layer (CRM to gene activity). No direct dependencies from first to third layer are allowed. A Bayesian Network was used to model the dependency between binding and CRM activity, while gene expression was assumed to be independently initiated by any active CRM within an acceptable range.

### Predicting CRM activity using a Bayesian Network

**Bipartite** Bayesian network was used to describe dependencies between TF binding and CRM activity. For each CRM, a quantitative binding score was computed for each of 15 TF/stage combinations (as previously described [48]) representing quantitative measurements for actual binary events of TF binding (Dataset S1). Each expression class (temporal or spatial) was represented by a separate binary variable. There were 5 temporal classes, representing stages 4–6, 7–8, 9–10, 11–12 and 13–16, following the BDGP nomenclature and 5 tissue-specific classes mesoderm (MESO), somatic muscle (SM), visceral muscle (VM), mesoderm and SM (MESO+SM), somatic and visceral muscle (SM+VM). Edges in the network represent dependencies of the conditional probability function of the variable corresponding to the CRM being active in a given condition on any variables representing TF binding events. Measured binding and activity for each CRM were considered to be a single observation from the same underlying joint distribution and they were used to find an optimal network. The network structure was constrained to only contain edges of this kind and probability distributions were optimized using BNfinder [24] software using Bayesian Dirichlet equivalence (BDe) score. No constraints on the resulting cpd function were set, however the binding signal was converted by the BNfinder software to probabilistic readouts of binary variable using a Gaussian mixture model. For detailed parameters used see Supplementary Text S1.

### Integrating CRM activity and chromatin data

All distances between a CRM and a transcriptional start site of a gene that were lower than 100 kb were tabulated based on FlyBase genome annotations, version 5.17 [49] (Dataset S2). For each CRM-promoter pair, the total number of insulator peaks was calculated in between them. Each gene is assumed to be able to respond to the activation signal from any of the paired CRMs, depending on the distance and the number of insulator peaks between them. It is assumed that the probability of activation by a CRM over a given distance *d* is linearly decreasing with *d* until it reaches *0* at the distance *dmax* or when the predefined limit of insulator peaks have been exceeded. Each promoter is assigned a probability of being activated in development based on the histone modification (H3K4me3) level measured within the 500 bp around the TSS, using non tissue-specific data (Dataset S4). For details see Supplementary text S1.

### Integrating different layers of the model using Expectation Maximization algorithm

The majority of CRMs (>95%) have unknown activity, so we treat all variables corresponding to CRM activity as latent and use a maximum likelihood principle to estimate them.

We define the likelihood function L
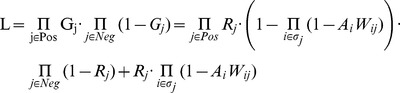
where G represents gene activity, i indexes CRMs, j indexes genes, Ai represents activity of the i-th CRM, Wij represents the weight of CRM-promoter interaction (depending on distance and insulators, as described earlier) and Rj representing the probability of a given promoter responding under specific conditions. Given this likelihood function we aim to find the most likely parameters of the model, i.e. the Bayesian Network and the optimal *dmax*. We use the Expectation Maximization (EM) strategy, by iteratively improving our current estimate of the parameters. Since the EM is a local optimization strategy, the result is highly dependent on the starting model. Normally this can be solved by starting from multiple randomized models, however in our case properly sampling a space of all Bayesian Networks would be difficult and likely to produce non-biological models. Instead we begin by initializing the BN parameters based on known CRM activity data (CAD [5]) by making the first inference not on the full training dataset but on the subset of the training set with experimentally measured tissue-specific activity. The *dmax* parameter could also have a strong impact in the initial stage of EM if it is set too low and therefore excludes some CRM data from the whole learning process. For this reason we initially set *dmax* to the maximum possible value and allow it to change freely from then on. The EM procedure is composed of alternating iteration of the expectation (E-step) and maximization (M-step) steps until convergence (improvement in the likelihood below 2%).

In the E-step, we calculate estimated probability of each CRM being active in each condition based on our current model parameters (BN and *dmax*). Since the model has three layers and we are interested in the estimation of the hidden variables from the middle one, we use an approach based on the forward-backward algorithm frequently used to infer the probabilities of the hidden variables in Hidden Markov Models [7]. In our case, the forward probability is the probability of the CRM being active given the TF binding data, and can be easily computed using the BN for all CRMs. The backward probability is the probability of the CRM being active given the gene expression data. We can ignore all genes *j* such that *wij = 0* as the change of the *i*-th CRM activity will not affect the total likelihood. For each CRM we need to consider all genes with *wij>0* and the CRM can only be inactive if each of the genes in its range is turned on by another CRM, which, by assumption of independent action of CRMs, can be computed using Bayes theorem and total probability. The overall activity of each CRM is determined by a smoothing step as the product of the respective forward and backward probabilities.

In the M-step, current estimates of CRM activity (the latent variables) are used for finding the model parameters (BN and *dmax*) maximizing the likelihood function L. For the BN, we are using the Bayesian Dirichlet equivalence optimization implemented in the BNfinder [24] library. Due to the constrained structure of the BN, it is possible to find a globally optimal network representing observed combinations of binding and activity very efficiently. As the likelihood function is not monotonous with respect to *dmax* we employed an exhaustive strategy to find the optimal *dmax* giving the maximum likelihood under assumed CRM activity and gene expression. This can be done quite efficiently with a step size of 200 bp, equal to the minimal size of the CRM.

The process was repeated until convergence; in the tested cases ∼10 iterations were required to reach improvement in one step below 2%. For a more detailed description see Supplementary text S1.

### Measuring predictive performance

For each expression class (temporal or spatial) the posterior probability calculated from the model was used as the ranking criteria to calculate the area under the curve (AUC) for the receiver operator characteristic (ROC) curve. The AUC value can be interpreted as the probability of a random positive example to have a higher posterior probability of expression than a random negative example. To assess the significance of the achieved AUC measures in comparison to random classifier or in comparison between different models we used the procedure proposed by Hanley and McNeil [50]. To avoid over-fitting, all models were trained in a 10-fold cross-validation scheme based on BDGP gene expression database release 2. Then the entire BDGP release 2 dataset was used for training the final models (Dataset S5), which were then tested on the gene expression patterns from BDGP release 3 (Dataset S6), excluding those from the training set. The same models were used to select genes from the visceral muscle activity class for validation by *in-situ* hybridization experiments. All training sets are available in Dataset S7.

### Software availability

The EM algorithm was implemented in Python using the BNfinder [24] library for estimating Bayesian networks, ROC curves were plotted with ROCR [51] package for R. All the scripts are available at https://code.launchpad.net/bnfinder/GEpredict

***In-situ*** **hybridizations** in *Drosophila* embryos were carried out using standard protocols as described previously [52]. The following ESTs from the *Drosophila* Gene Collection (DGC) were used to generate Digoxigenin-labeled probes: GM02640 (*Eip75B*), LD09907 (*Hex-A*), RE05370 (*CG9194*), GM10074 (*bt*), AT24194 (*Rya-r44f*), LP05734 (*Hsp22*), GH06348 (*CG1516*), RH17388 (*CG10654*), GH24653 (*A3-3*), SD01953 (*by*), LP03829 (*CG6981*), GH27027 (*Ncc69*), SD11716 (*CG14709*), HL01392 (*fau*), LP06027 (*Cpr78E*), GH06222 (*CG13124*), LD02379 (*nrv1*), RE70568 (*Lim3*), LD44720 (*CG7530*), GH23506 (*CG14655*), LP04481 (*CG6770*), GH19382 (*CG4945*). *biniou* cDNAs (from M. Frasch) was used to generate Biotin-labeled probe. Double *in-situs* hybridizations were performed by using anti-Digoxigenin-POD and anti-Biotin-POD antibodies (Roche) and detected sequentially with FITC and Cy3 (Perkin-Elmer TSA kit). A Zeiss LSM 510 confocal microscope was used for imaging.

## Supporting Information

Dataset S1 **Quantitative TF occupancy for 8008 CRMs and 15 different ChIP experiments.**
(XLS)

Dataset S2 **Distances (<100 000) between transcriptional start sites of 14689 genes and all 8008 CRMs including count of insulator peaks in-between them.**
(XLS)

Dataset S3 **CRM activity from CAD database in spatial (a) and temporal (b) classes.**
(XLS)

Dataset S4 **Promoter activity estimates based on histone H3K4 tri-methylation from mod-encode for spatial (a) and temporal (b) expression classes.**
(XLS)

Dataset S5 **Gene activity, based on in-situ annotations, for spatial (a) and temporal (b) classes – training data.**
(XLS)

Dataset S6 **New annotations of gene activity based on BDGP release 3 (2010). In spatial (a) and temporal (b) classes.**
(XLS)

Dataset S7 **Predictions for all genes and all classes made by the full model trained with the EM procedure.**
(XLS)

Dataset S8 **Manually curated annotations for top 100 VM predictions.**
(XLS)

Figure S1 **Examples showing the general complexity of gene loci and the difficulty in linking CRMs to their appropriate target gene.** Genomic regions for tinman+bagpipe (**a**), CG6981 (**b**) and Fas3 (**c**). Depicted tracks represent, from top to bottom: Transcription factor binding (ChIP signal shown for one of 15 developmental conditions in blue), CP190 insulator binding (ChIP signal shown for one of 6 factors in red), Histone H3 K4 tri-methylation for a selected time-point (orange). ChIP defined mesodermal CRM locations are indicated by blue rectangles and gene models from refseq are indicated in black. All loci contain inactive genes (no histone mark) very close to bound CRMs. These ‘bystander’ genes are often surrounded by CRMs from neighboring genes (**a**), can contain an active gene within their own intron (**b**) or are in an intron of an active gene (**c**).
(TIFF)

Figure S2 **Predicting gene expression based on a simple additive model summing multiple CRM activities in the vicinity of the closest gene.** Predicting gene expression based on an SVM model optimized for CRM activity (Zinzen *et al.* (2009)). The SVM provides numerical classification for all CRMs in 5 classes. Each CRM was assigned to the closest gene. For each gene, the sum of prediction values of all assigned CRMs represents the prediction value for the gene for each activity class. The results are presented as Receiver operator curves (ROC) for all 5 expression classes published by Zinzen *et al.* The area under the curve (AUC) is given for each class. Meso_only = genes with expression in unspecified mesoderm, but not in derived muscle tissue; SM_only = genes with expression in the somatic muscle, but not in the mesoderm or other muscle tissues; VM_only = genes with expression in visceral muscle and not in the mesoderm or other muscle tissues; VM_SM = genes with expression in both the visceral muscle and somatic muscle, and not in the early mesoderm; meso_SM = genes with expression in the early mesoderm and somatic muscle, and not in visceral muscle.
(TIFF)

Figure S3 **Comparison of predictions by BNs and SVMs.** Performance comparison between SVM-based model and full probabilistic model at the gene (**a**) and CRM (**b**) level. For all five activity classes for which the SVM model was trained by Zinzen *et al*, we provide a gene-based AUC value for SVM ((**a**), blue bars) and the proposed model ((**a**), red bars). Panel (**b**) shows the overlaid ROC curves for the SVM model (yellow-green-blue curve) and the Bayesian network (red curve) resulting from the iterative learning procedure. Even though the Bayesian model was not explicitly optimizing performance of CRM predictions, it provides comparable results (b). For gene activity predictions (a), the BN model clearly outperforms the SVM.
(TIFF)

Figure S4 **Performance of the gene expression prediction using the full iterative probabilistic model.** Performance of the full model for each class is represented by a ROC curve. Corresponding activity class and the area under the curve (AUC) is presented in the title for each graph. Color coding (y-axis) represents the posterior probabilities of activity estimated by the model, ranging from red (most probable) to blue (least probable). 13 activity classes were examined. 8 spatial classes: meso, SM, VM, meso_only, SM_only, VM_only, meso_SM, VM_SM and 5 temporal classes: developmental stages 4–6, stages 7–8, stages9–10, stages 11–12, stages 13–16. Meso = mesoderm; SM = somatic muscle; VM = visceral muscle; Meso_only = genes with expression in unspecified mesoderm, but not in derived muscle tissue; SM_only = genes with expression in the somatic muscle, but not in the mesoderm or other muscle tissues; VM_only = genes with expression in visceral muscle and not in the mesoderm or other muscle tissues.
(TIFF)

Figure S5 **Performance comparison between cross-validated and full model.** ROC curves corresponding to each of 10 cross-validations (grey), their average (black) and the ROC curve corresponding to non-cross-validated data (blue). The performance of non-cross-validated model does not differ significantly from the average of cross-validated models suggesting that the non-cross-validated model is not overfitting.
(TIFF)

Figure S6 **Performance comparison with simpler models.** Average performance, as measured by average AUC for all activity classes, is shown for the full model (TF+INS+HIST+EM) in comparison with different simplified versions, SVM-based additive model using the closest gene and a random classifier. TF = transcription factor occupancy; INS = insulator binding; HIST = histone modification (H3K4me3) marking active promoters; EM = expectation maximization used for the iterative full model.
(TIFF)

Figure S7 **Gene expression prediction using a 2-layer model.** ROC curves describing performance of a 2-layer model (black lines) computing the probability of gene activity based on a Bayesian Network mapping TF binding data directly to gene expression (without the intermediate CRM activity layer). Blue and black plots correspond to the predictions made with the full and 2-layer model, respectively.
(TIFF)

Figure S8 **Validation using expression data for 600 new genes.** Comparison of AUC measures of predictions from the model trained on BDGP 2007 as measured on the training set (blue) and the new genes, annotated in BDGP 2010 that were not present in the training set. Classes containing less than 5 genes in the validation positive set were removed. The remaining classes are meso = mesoderm; sm = somatic muscle; vm = visceral muscle; meso_only = genes with expression in unspecified mesoderm, but not in derived muscle tissue; developmental stages 7–8; stages 9–10; stages 11–12 and stages 13–16.
(TIFF)

Figure S9 **Validating spatio-temporal expression predictions in the visceral muscle.** Embryo images showing double fluorescent *in-situ* hybridization against the gene with predicted visceral muscle (VM) expression (red) and a specific marker for VM (green), where overlapping gene expression in VM is shown in the merge panel. The 11 genes in panel (**a**) are expressed in VM, indicated by the white arrows. While the genes in panel (**b**) are not expressed in VM, they are expressed at the predicted stages of development and are typically expressed in a VM related tissue (e.g. the midgut in the case of *CG6981* and *CG6770*). All embryos are orientation with anterior to the left and dorsal up.
(TIFF)

Figure S10 **Performance of the model without the initially supplied CRM activity data.** For each of the three largest tissue-specific expression classes the model was initialized with CRM activity based on the nearest gene expression data supplied instead of the actual CRM activity. Grey lines represent the ROC curves for all 10 folds in the cross-validation scheme, the color line represents the performance of the model trained from all data. Area under the curve is reported for the full model. For comparison of these AUCs with the cross-validated three-layer optimized model (Supple Fig. S4) is as follows: Optimized model- Meso AUC = 0.819; optimized model-SM AUC = 0.797; optimized model-VM AUC = 0.878.
(TIFF)

Figure S11 **Number of CRMs per gene (A) and number of genes per CRM (B) plotted as a function of maximum distance of target gene assignment.** The plots show the average number of CRMs per gene (A) or genes per CRM (B) plotted as a function of the maximum distance used in the gene-CRM assignment function. Depending on whether insulator peaks are used to limit the number of assignments, either a linear growth of the number of assignments (no insulators) or a saturation at distances above 50 kbp are obtained. It should be noted that when using insulator data, there is no need to search further than approximately 50 kbp as virtually all CRMs beyond this point are already insulated from their potential targets, i.e. the weight of their activity influence on the gene's expression (w_ij_) is equal 0.
(TIFF)

Table S1 **List of all used datasets with references.**
(XLS)

Text S1 **Supplementary** **Methods** **.** The Model: The text describes a general overview of the layered structure of the model, followed by a detailed description of the three layers (layer 1-TF binding, layer 2-CRM activity, layer 3- gene activity. This is followed by a description of how these three layers are integrated using iterative optimization. A simplified model: The text describes the implementation and performance of a two-layer model (without known CRM activity data). The final section of the supplementary methods describes the statistical analysis of Gene-CRM assignment.
(DOC)
