# Supplementary figures and images for: Predicting Spatial and Temporal Gene Expression Using an Integrative Model of Transcription Factor Occupancy and Chromatin State
Source: PLoS Comput Biol. 2012 Dec 6;8(12):e1002798. doi: 10.1371/journal.pcbi.1002798 (PMC3516547; doi:10.1371/journal.pcbi.1002798)

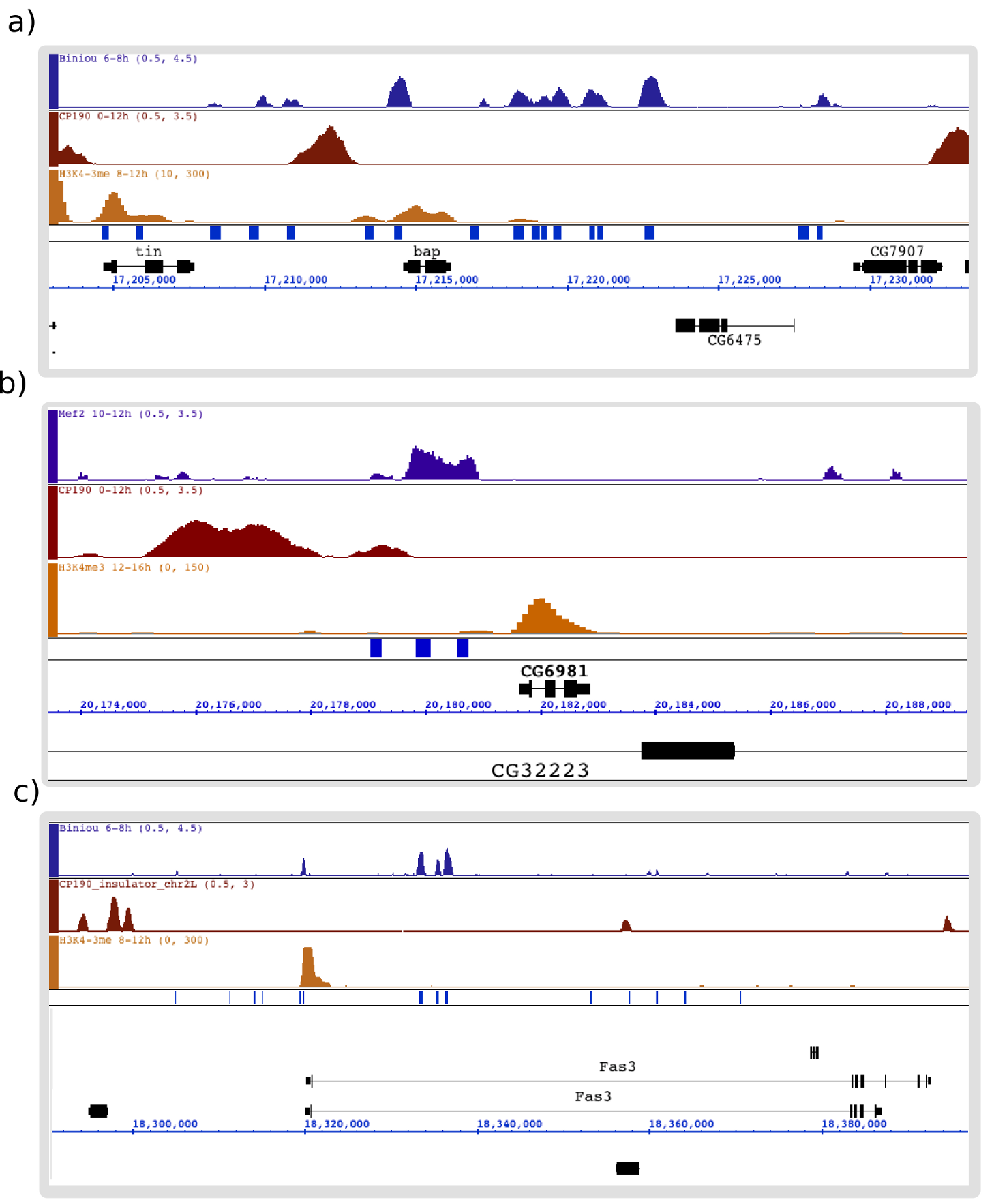

Supplement: Figure S1 — Examples showing the general complexity of gene loci and the difficulty in linking CRMs to their appropriate target gene. Genomic regions for tinman+bagpipe (a), CG6981 (b) and Fas3 (c). Depicted tracks represent, from top to bottom: Transcription factor binding (ChIP signal shown for one of 15 developmental conditions in blue), CP190 insulator binding (ChIP signal shown for one of 6 factors in red), Histone H3 K4 tri-methylation for a selected time-point (orange). ChIP defined mesodermal CRM locations are indicated by blue rectangles and gene models from refseq are indicated in black. All loci contain inactive genes (no histone mark) very close to bound CRMs. These ‘bystander’ genes are often surrounded by CRMs from neighboring genes (a), can contain an active gene within their own intron (b) or are in an intron of an active gene (c). (TIFF) [file pcbi.1002798.s009.tiff]

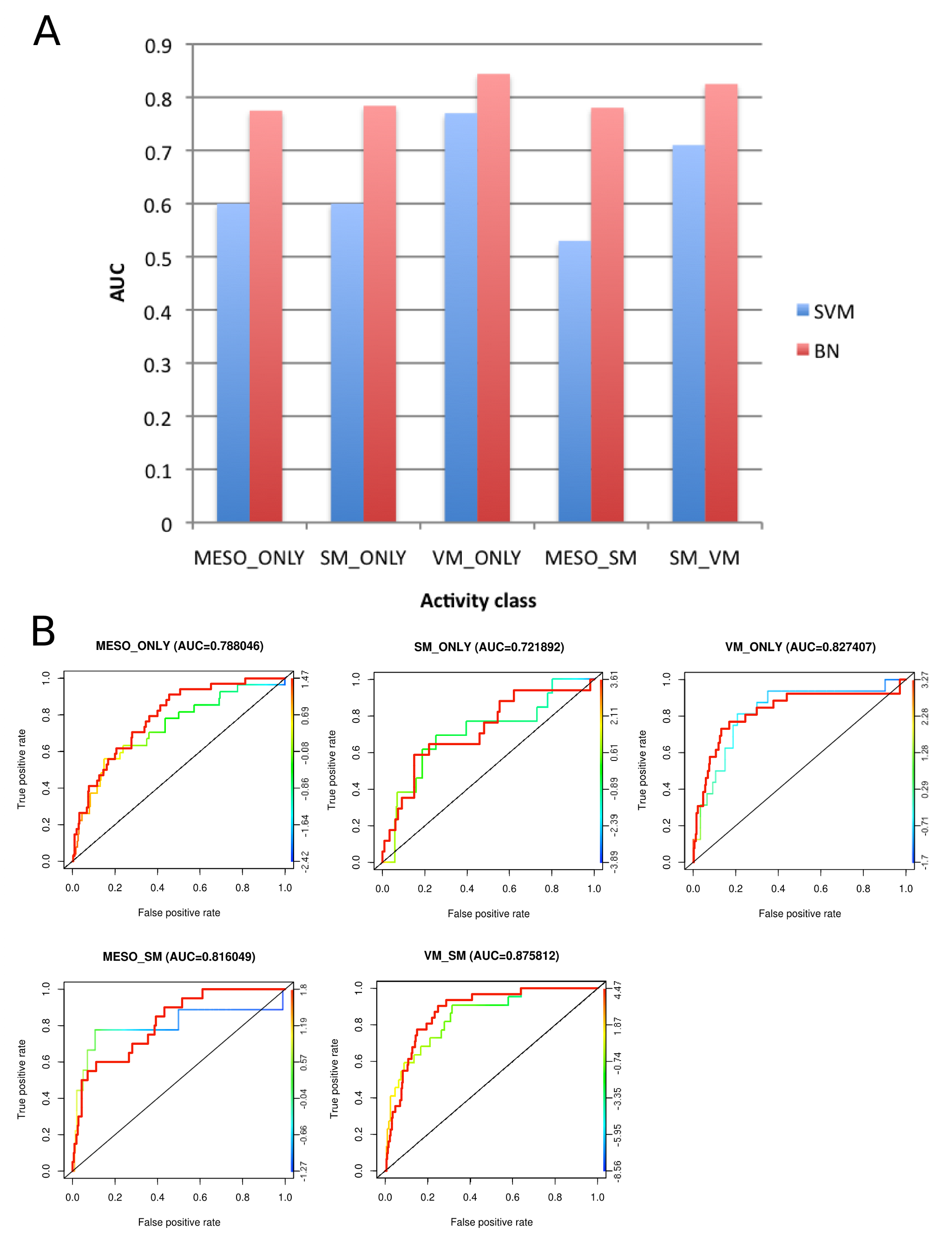

Supplement: Figure S3 — Comparison of predictions by BNs and SVMs. Performance comparison between SVM-based model and full probabilistic model at the gene (a) and CRM (b) level. For all five activity classes for which the SVM model was trained by Zinzen et al, we provide a gene-based AUC value for SVM ((a), blue bars) and the proposed model ((a), red bars). Panel (b) shows the overlaid ROC curves for the SVM model (yellow-green-blue curve) and the Bayesian network (red curve) resulting from the iterative learning procedure. Even though the Bayesian model was not explicitly optimizing performance of CRM predictions, it provides comparable results (b). For gene activity predictions (a), the BN model clearly outperforms the SVM. (TIFF) [file pcbi.1002798.s011.tiff]

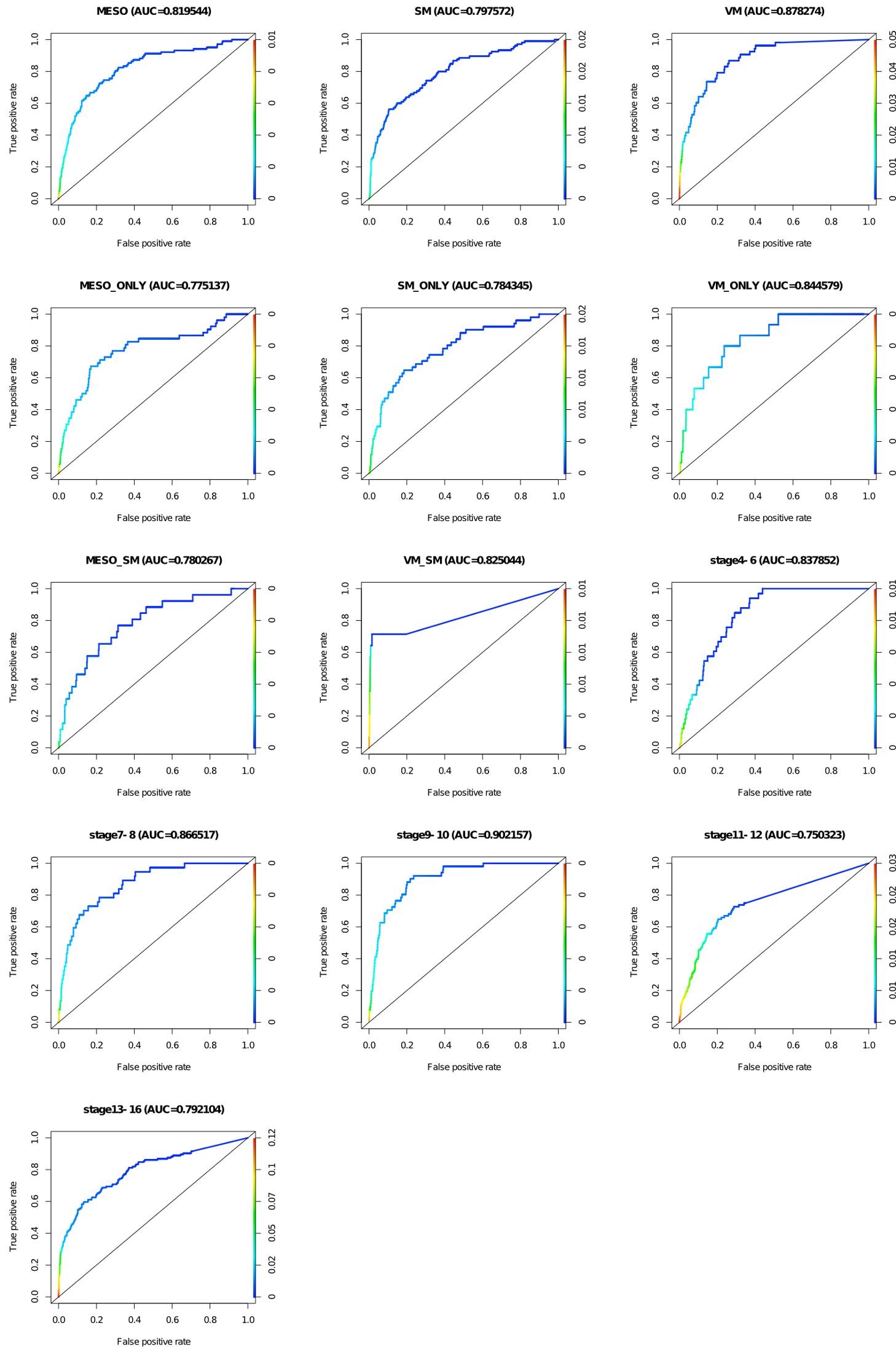

Supplement: Figure S4 — Performance of the gene expression prediction using the full iterative probabilistic model. Performance of the full model for each class is represented by a ROC curve. Corresponding activity class and the area under the curve (AUC) is presented in the title for each graph. Color coding (y-axis) represents the posterior probabilities of activity estimated by the model, ranging from red (most probable) to blue (least probable). 13 activity classes were examined. 8 spatial classes: meso, SM, VM, meso_only, SM_only, VM_only, meso_SM, VM_SM and 5 temporal classes: developmental stages 4–6, stages 7–8, stages9–10, stages 11–12, stages 13–16. Meso = mesoderm; SM = somatic muscle; VM = visceral muscle; Meso_only = genes with expression in unspecified mesoderm, but not in derived muscle tissue; SM_only = genes with expression in the somatic muscle, but not in the mesoderm or other muscle tissues; VM_only = genes with expression in visceral muscle and not in the mesoderm or other muscle tissues. (TIFF) [file pcbi.1002798.s012.tiff]

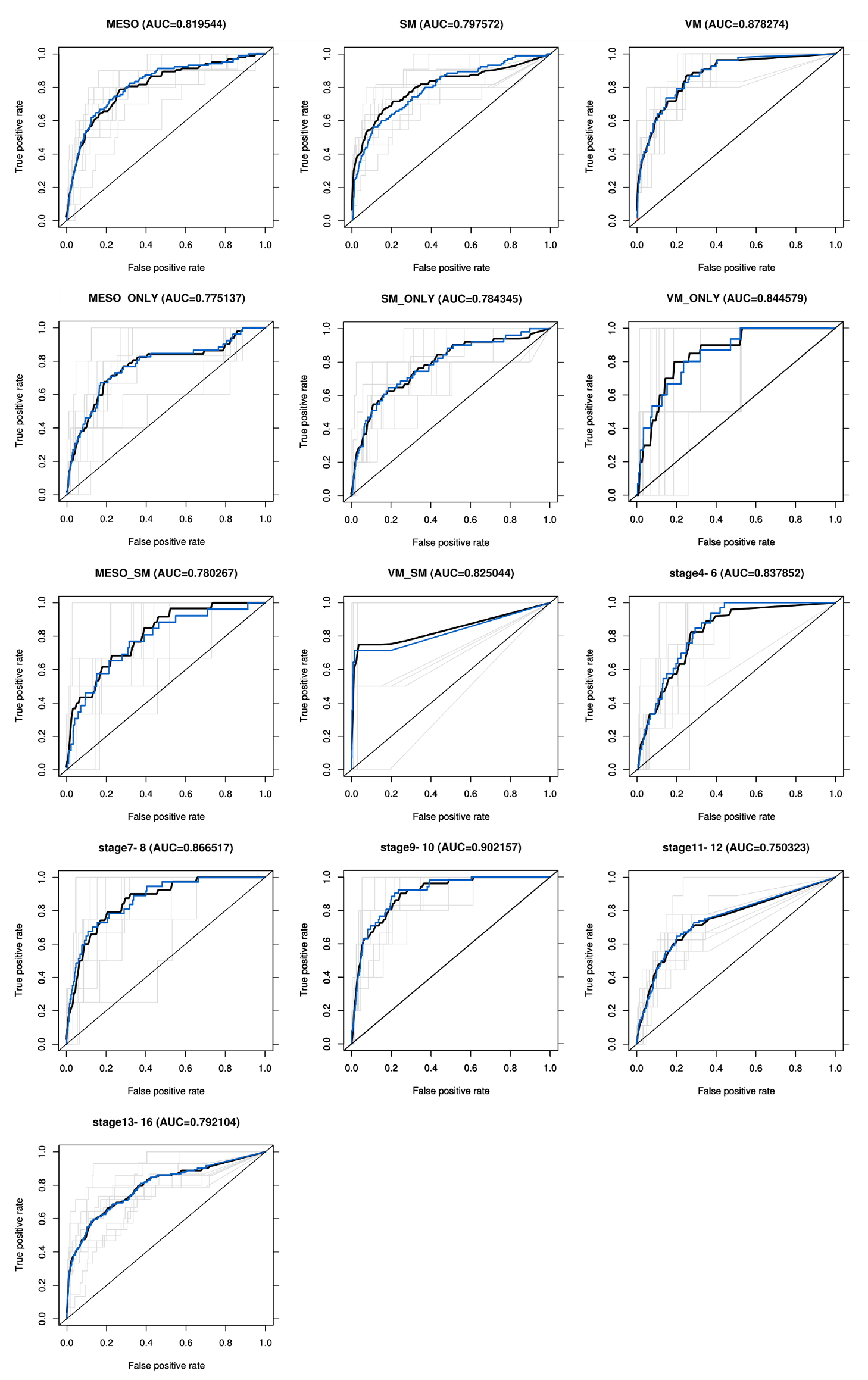

Supplement: Figure S5 — Performance comparison between cross-validated and full model. ROC curves corresponding to each of 10 cross-validations (grey), their average (black) and the ROC curve corresponding to non-cross-validated data (blue). The performance of non-cross-validated model does not differ significantly from the average of cross-validated models suggesting that the non-cross-validated model is not overfitting. (TIFF) [file pcbi.1002798.s013.tiff]

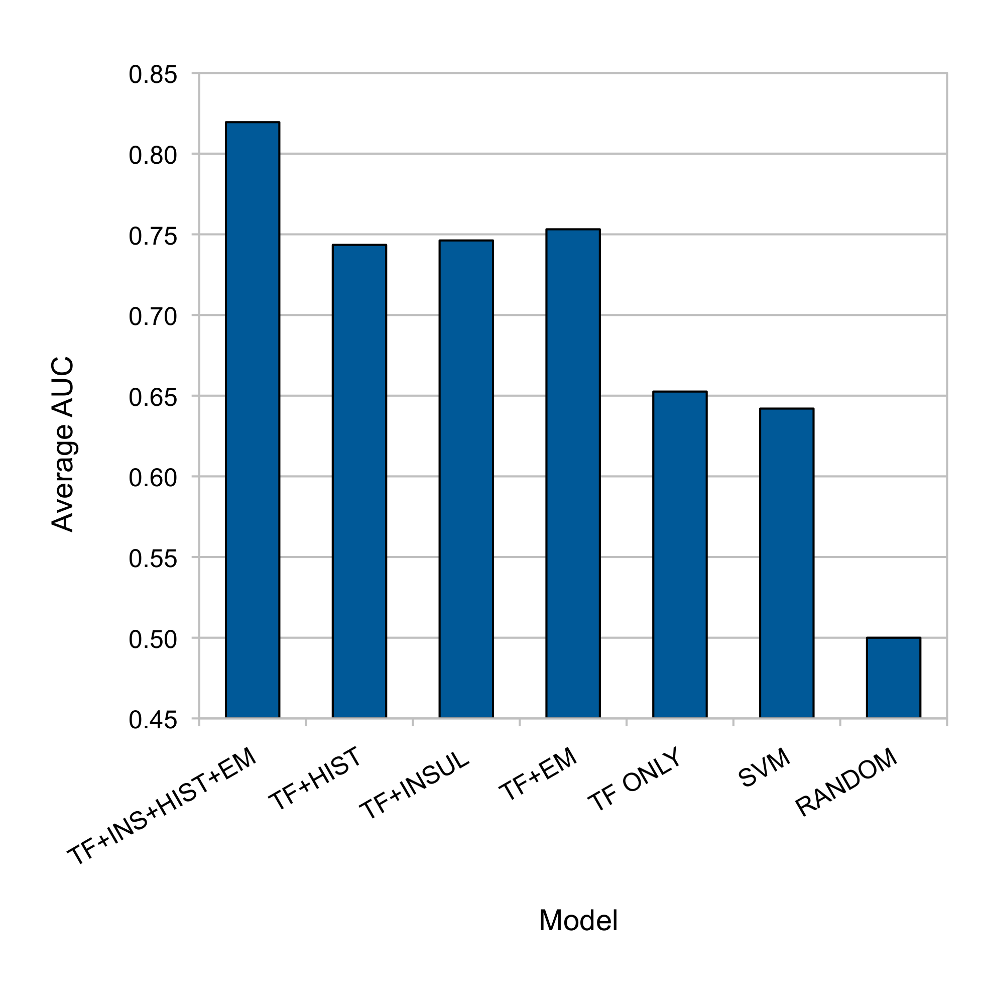

Supplement: Figure S6 — Performance comparison with simpler models. Average performance, as measured by average AUC for all activity classes, is shown for the full model (TF+INS+HIST+EM) in comparison with different simplified versions, SVM-based additive model using the closest gene and a random classifier. TF = transcription factor occupancy; INS = insulator binding; HIST = histone modification (H3K4me3) marking active promoters; EM = expectation maximization used for the iterative full model. (TIFF) [file pcbi.1002798.s014.tiff]

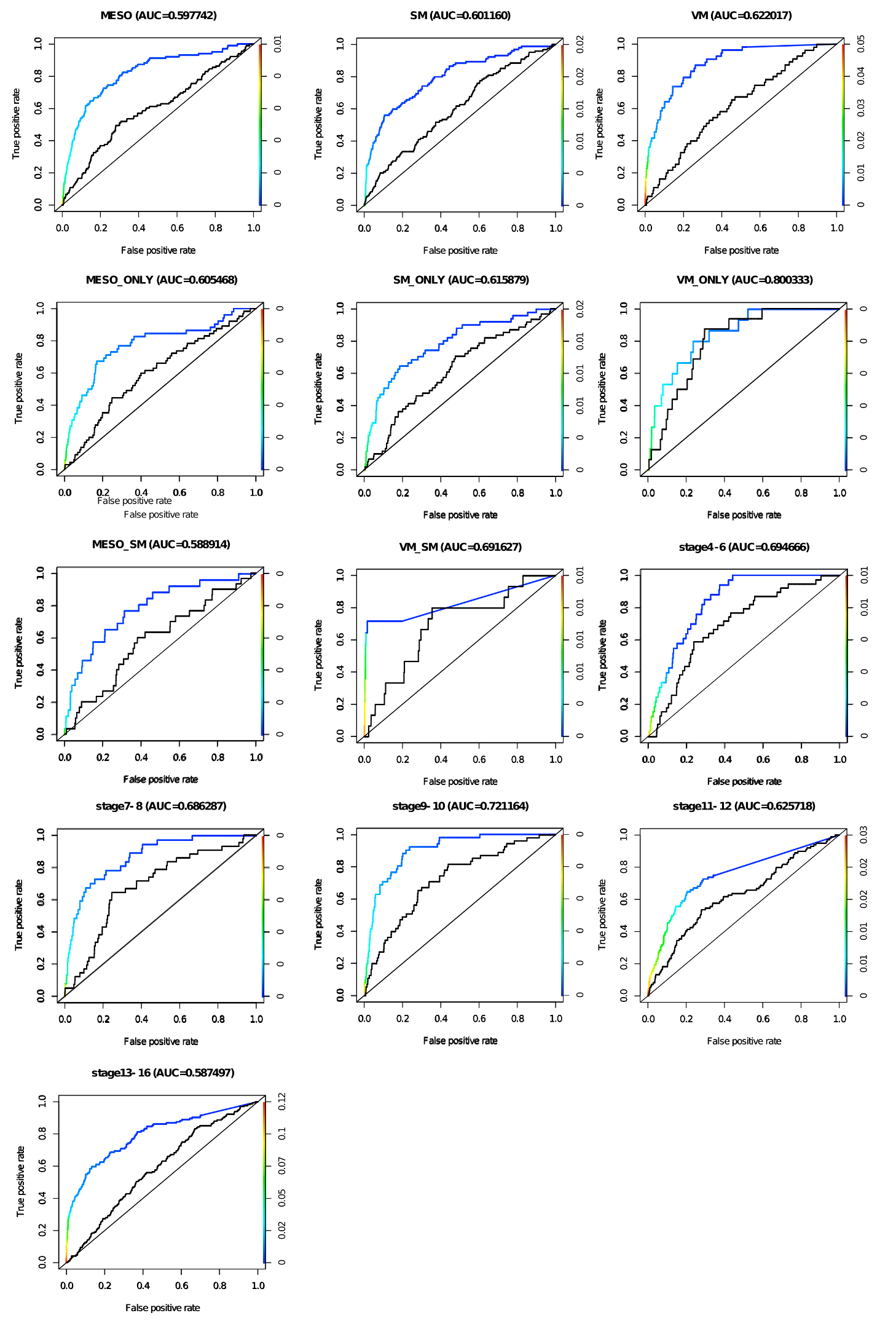

Supplement: Figure S7 — Gene expression prediction using a 2-layer model. ROC curves describing performance of a 2-layer model (black lines) computing the probability of gene activity based on a Bayesian Network mapping TF binding data directly to gene expression (without the intermediate CRM activity layer). Blue and black plots correspond to the predictions made with the full and 2-layer model, respectively. (TIFF) [file pcbi.1002798.s015.tiff]

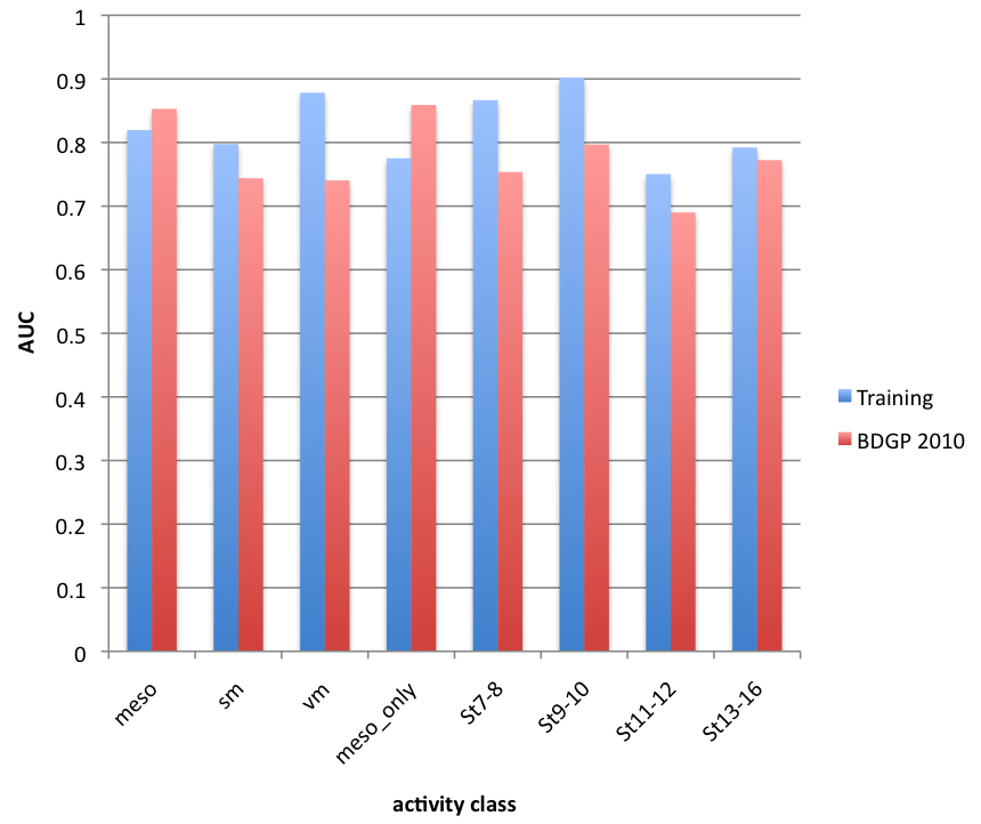

Supplement: Figure S8 — Validation using expression data for 600 new genes. Comparison of AUC measures of predictions from the model trained on BDGP 2007 as measured on the training set (blue) and the new genes, annotated in BDGP 2010 that were not present in the training set. Classes containing less than 5 genes in the validation positive set were removed. The remaining classes are meso = mesoderm; sm = somatic muscle; vm = visceral muscle; meso_only = genes with expression in unspecified mesoderm, but not in derived muscle tissue; developmental stages 7–8; stages 9–10; stages 11–12 and stages 13–16. (TIFF) [file pcbi.1002798.s016.tiff]

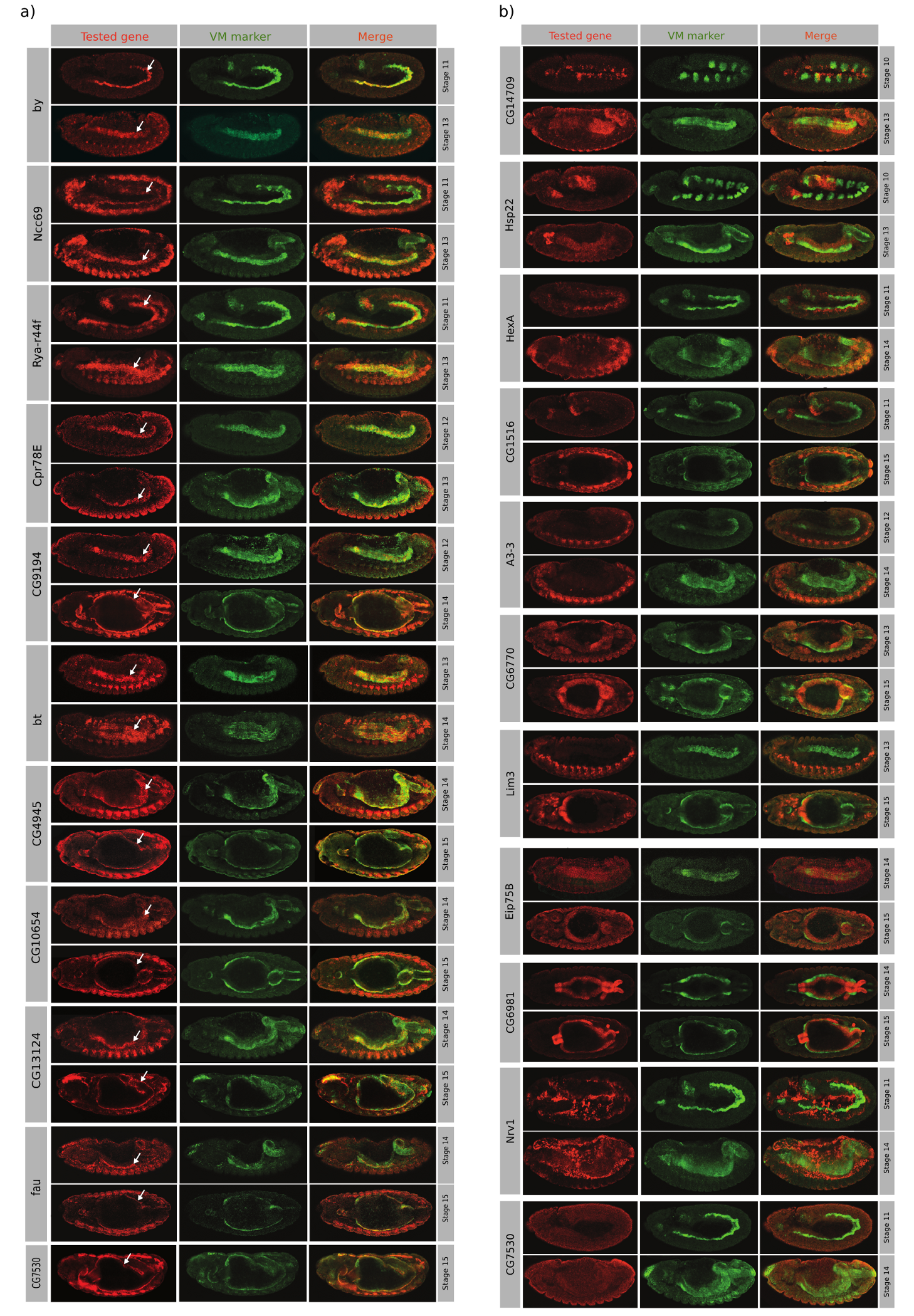

Supplement: Figure S9 — Validating spatio-temporal expression predictions in the visceral muscle. Embryo images showing double fluorescent in-situ hybridization against the gene with predicted visceral muscle (VM) expression (red) and a specific marker for VM (green), where overlapping gene expression in VM is shown in the merge panel. The 11 genes in panel (a) are expressed in VM, indicated by the white arrows. While the genes in panel (b) are not expressed in VM, they are expressed at the predicted stages of development and are typically expressed in a VM related tissue (e.g. the midgut in the case of CG6981 and CG6770). All embryos are orientation with anterior to the left and dorsal up. (TIFF) [file pcbi.1002798.s017.tiff]

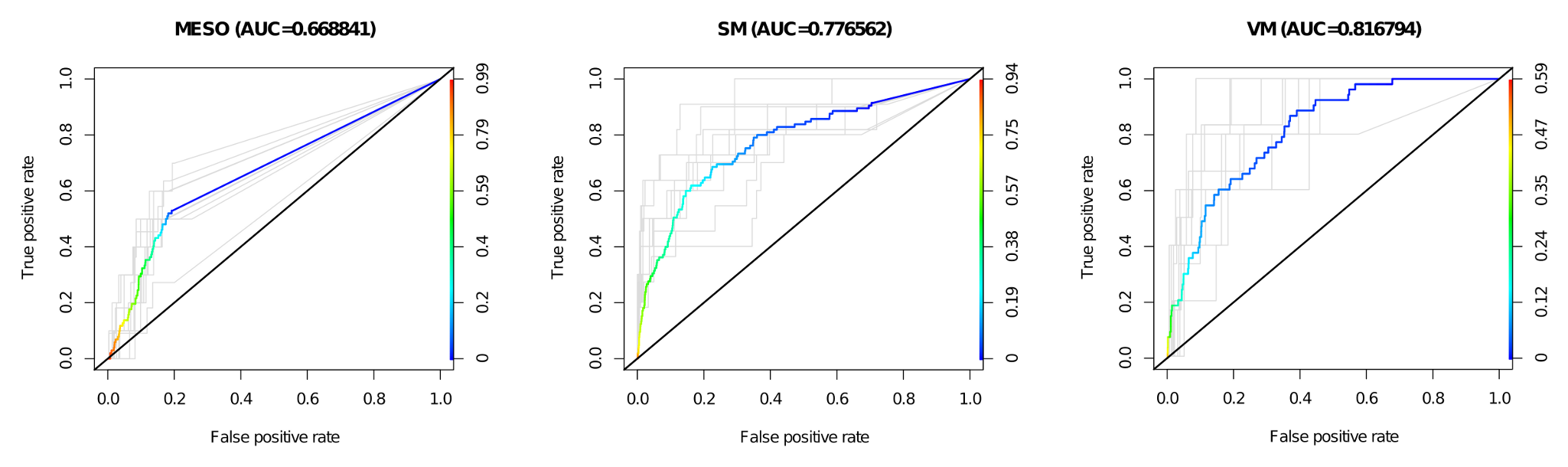

Supplement: Figure S10 — Performance of the model without the initially supplied CRM activity data. For each of the three largest tissue-specific expression classes the model was initialized with CRM activity based on the nearest gene expression data supplied instead of the actual CRM activity. Grey lines represent the ROC curves for all 10 folds in the cross-validation scheme, the color line represents the performance of the model trained from all data. Area under the curve is reported for the full model. For comparison of these AUCs with the cross-validated three-layer optimized model (Supple Fig. S4) is as follows: Optimized model- Meso AUC = 0.819; optimized model-SM AUC = 0.797; optimized model-VM AUC = 0.878. (TIFF) [file pcbi.1002798.s018.tiff]

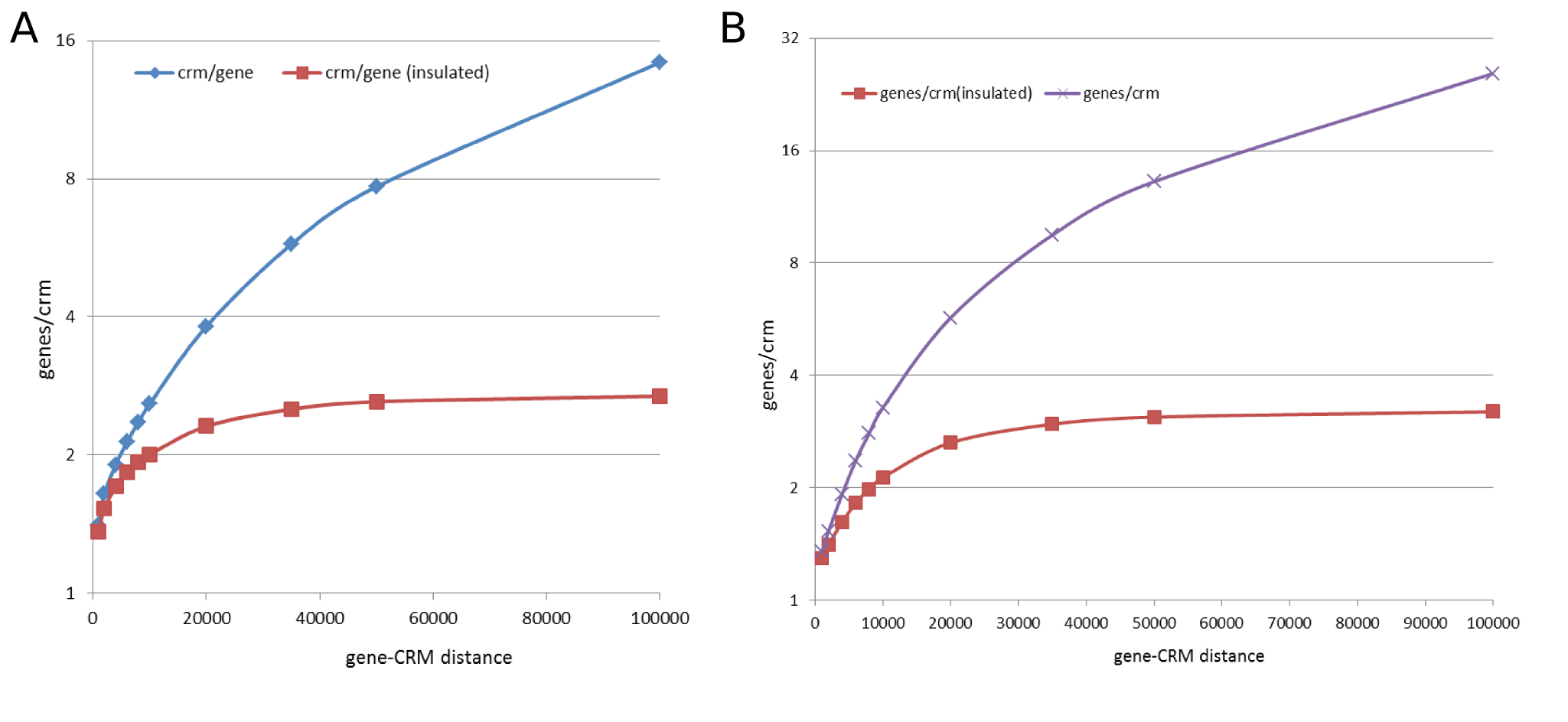

Supplement: Figure S11 — Number of CRMs per gene (A) and number of genes per CRM (B) plotted as a function of maximum distance of target gene assignment. The plots show the average number of CRMs per gene (A) or genes per CRM (B) plotted as a function of the maximum distance used in the gene-CRM assignment function. Depending on whether insulator peaks are used to limit the number of assignments, either a linear growth of the number of assignments (no insulators) or a saturation at distances above 50 kbp are obtained. It should be noted that when using insulator data, there is no need to search further than approximately 50 kbp as virtually all CRMs beyond this point are already insulated from their potential targets, i.e. the weight of their activity influence on the gene's expression (wij) is equal 0. (TIFF) [file pcbi.1002798.s019.tiff]
