# Supplementary material for: Predicting Spatial and Temporal Gene Expression Using an Integrative Model of Transcription Factor Occupancy and Chromatin State
Source: PLoS Comput Biol. 2012 Dec 6;8(12):e1002798. doi: 10.1371/journal.pcbi.1002798 (PMC3516547; doi:10.1371/journal.pcbi.1002798)
Supplement: Text S1 — Supplementary Methods . The Model: The text describes a general overview of the layered structure of the model, followed by a detailed description of the three layers (layer 1-TF binding, layer 2-CRM activity, layer 3- gene activity. This is followed by a description of how these three layers are integrated using iterative optimization. A simplified model: The text describes the implementation and performance of a two-layer model (without known CRM activity data). The final section of the supplementary methods describes the statistical analysis of Gene-CRM assignment. (DOC) [file pcbi.1002798.s021.doc]

# Supplementary Text S1:

## Predicting spatio-temporal gene expression using a integrative model of transcription factor occupancy and chromatin state

Bartek Wilczynski, Ya-Hsin Liu, Zhen Xuan Yeo and Eileen EM Furlong

# Supplementary Methods:

## General overview of the layered structure of the model

Our aim is to build a model describing the probability of any given gene being transcriptionally active in certain conditions (tissues or stages) of development. The model uses data on transcription factor (TF) occupancy, insulator binding and chromatin modifications to predict predefined patterns of expression as observed by in-situ hybridization experiments. Guided by our knowledge of the underlying biological process, we have structured the model into three layers, each of which consists of random variables representing a certain type of events impinging on the process of transcriptional regulation.

The first layer represents binding of TFs to 8008 *cis*-regulatory elements obtained from ChIP-Chip assays of 5 different TFs at 5 developmental stages [1], the second layer describes activity of the 8008 CRMs categorized by temporal and spatial activity classes, and the third layer corresponds to transcriptional activity of all *Drosophila* genes in the same activity classes as CRMs. We assume that the events follow a natural sequence from TF binding through CRM activity to the initiation of gene transcription, however the exact course of action might be different in different tissues or stages. In a probabilistic framework, this is described by the probability of a gene being active in a certain condition as a dependant on the activity of neighboring CRMs in the same condition. The CRM activity, in turn, depends on TF binding. Importantly, there can be no shortcuts or horizontal flow of information in the model, i.e. no CRM activity depends directly on another CRM’s activity and no TF binding events can affect gene activity other than through activating their targeted CRMs. Gene activity is, however, also dependent on the local state of chromatin, i.e. the level of H3K4-tri-methylation at the promoter, the distance of CRMs to the transcriptional start site and the relative location of CRMs and insulator proteins.

We first describe variables constituting all three layers of the model and then focus on the way our probabilistic model encodes the relations between them. An overview of the different layers of the model and their interconnections is provided in Figure 1b of the main text.

## Layer 1 - TF binding

Zinzen et al [1] performed 15 ChIP-Chip experiments in *Drosophila* embryos to elucidate the transcriptional network regulating mesoderm development. They assayed 5 TFs (Twist, Tinman, Biniou, Bagpipe and Mef2) that are crucial for the proper development of mesoderm across 5 different stages of development, appropriate for the expression of the respective factors. Then, by clustering the discovered ChIP signal peaks, they compiled an atlas of putative *cis*-Regulatory sequences bound with high confidence by at least one TF at one time-point. Thirty-six of these ChIP-defined CRMs were experimentally validated in transgenic embryos, which demonstrated that over 97% are able to function as regulatory elements *in vivo*.

The binary nature of peaks defined by thresholding ChIP signal makes them an imperfect description of TF binding, especially for estimation of a probabilistic model. We have shown previously [2] that a maximum average from a sliding window of 200bp over each CRM is a more accurate method to obtain quantitative signals extracted from ChIP, and revealed temporal binding patterns between CRMs. In the current study, we employ the same approach to extract quantitative signal, however we further process it to obtain probabilities of TF binding. Namely, for each condition (e.g. twist binding at 2-4h) we extract quantitative signal for all CRMs as described previously [2]. The distribution of such signals represent a mixture of noisy signals coming from bound and unbound CRMs. Indeed, this distribution for all conditions can be described by a mixture of two Normal distributions, although the mean and variance of the bound CRM signals is quite variable. After estimating parameters of the mixture for each condition, we obtain a likelihood of each CRM being really bound in each experiment. This gives a Matrix B *= {B_im_},* where *i*=1..8008 represents different CRMs and *m=*1..15 corresponds to different conditions. Each variable *b_im_* describes the estimated probability that the observed quantitative signal for crm *i* was generated from a bound TF at condition *m.*

## Layer 2 - CRM activity

The second model layer corresponds to CRM activity. For each CRM mentioned in the previous section, we describe its activity in:

5 temporal stages:

- stage 4-6,
- stage 7-8,
- stage 9-10,
- stage 11-12,
- stage 13-16 and

5 spatial classes:

- mesoderm (MESO),
- somatic muscle (SM),
- visceral muscle (VM),
- mesoderm and SM (MESO+SM),
- somatic and visceral muscle (SM+VM).

Additionally, each of the simple classes (MESO, SM, VM) are also considered in their exclusive (not expressed in other tissues) or non-exclusive variants. As it is difficult to measure CRM activity as a quantitative output *in vivo*, we describe CRM activity as a matrix A of binary variables A_ik_ , representing just two levels of activity: active/inactive in a given activity class, independently for spatial and temporal classes. While *i* indexes CRMs, the new variable k corresponds to one of the 10 (or 13 if we count exclusive classes separately) activity classes. Each variable A_ik_ represents the probability of CRM *i* being active in condition k. Importantly, as *in vivo* enhancer validation is a laborious process, it is difficult to obtain a large number of tested CRMs for training. The mesoderm-muscle system during *Drosophila* development represents one of the best studied systems, with several hundred CRMs tested for activity *in vivo* (in the CRM-activity database: CAD[1]). There is also accumulating transgenic data for enhancer activity in mice and fish. However, to make the method applicable to systems where the activity of only a few enhancers have been examined *in vivo*, we treat enhancer activity as hidden, unobservable variables, which can be only estimated using the maximum likelihood principle from other data. The experimental data from CAD[1] is used only for initiation step of the iterative estimation procedure.

The activity of the vast majority of the 8008 ChIP-CRMs is unknown. To estimate their activity we define a conditional probability of a CRM being active depending on its binding pattern P(A_ik_|B_1_..B_15_). While theoretically we could estimate this probability directly from data, there is a complexity problem with the fact that the number of possible binding patterns (2^15=32768) is higher than the number of CRMs, meaning that the estimation problem is greatly underdetermined. However, we do not expect all binding events to influence every activity class. For this reason, we use Bayesian networks[6] to describe conditional probability of CRM activity. In such a network, each activity class and each binding event is represented by a node in the network. These nodes are connected by an edge <k,m> only in the case when the probability of activity in class k depends on binding event m. Given the matrices A and B we can find the network structure optimal with respect to the Bayesian Dirichlet equivalence (BDe) criteria, optimizing the Bayesian likelihood of observing the matrices A and B given such network. The BDe criterion has several important features, making it suitable for our application: it automatically penalizes for overly complex network structures by integrating over all possible probability distributions for a given topology and an implementation of an efficient algorithm for finding such networks is available in the BNfinder package [7]. We have incorporated the BNfinder library into custom software package, and made it freely available as open source library. To avoid over-fitting to a single network model, the method uses BNfinder option of calculating scores of 10 best models and combining edges receiving at least 5% of the likelihood to create the final network. Once such a network is found, it can be used as a formal description of a conditional probability distribution, which can be used to calculate the probability of CRM activity given the probabilities of all relevant binding events. In addition, its edges can be interpreted as a measure of the importance of different TFs in the determination of certain expression patterns.

## Layer 3 - Gene activity

The third layer corresponds to spatio-temporal expression of genes in the same activity classes as in the previous step. Similar to CRMs, genes’ activity is described by binary variables, again largely due to the non-quantitative nature of the data. Even though there are a number of microarray datasets available for quantitative gene expression profiling during *Drosophila* development[3, 4], they are obtained from whole embryos making them unsuitable for our purpose of modeling tissue specific regulation. In contrast, binary annotation of gene activity in spatial and temporal resolution can be obtained for 5992 genes from the BDGP in-situ expression database[5] and predictions for un-annotated genes can be readily tested by in-situ hybridization. Thus, we model gene activity as a matrix G ={G_jk_}, where j corresponds to the gene index and k to the activity class. Again, the coverage of available data for gene activity is not complete, however it is already close to 45% of all *Drosophila* genes, so instead of treating them as hidden variables, we simply use only the set of genes for which we have expression data to estimate the model.

In order to estimate the probability of a given gene being activated under certain conditions, a number of different datasets need to be integrated: (i) the CRM activity estimates from matrix A, (ii) the relative distance between the promoter of the gene and each CRM (within an expansive region of 50k bp), (iii) the state of the chromatin at the promoter and (iv) the presence of insulators between the CRM and the promoter.

To help to identify with gene’s promoters are activated by specific developmental enhancers[8], we use histone modification data from the ModEncode project to estimate probabilities (R_j_) of gene j being activated in a given condition. This probability is dependent on the state of hitone H3 lysine-4 3-methylation, as measured in the ModEncode project at three time points 4-8h, 8-12h, 12-16h roughly corresponding to the stages of mesoderm and muscle development. The conditional probability distribution for R_j_ is constructed using a BN model based on discretized histone data (similar to the CRM activity model).

Although there are reported cases of long-range enhancers (CRMs) in *Drosophila*, these are currently the rare exceptions. The vast majority of known CRMs are located within 5kb upstream or downstream of the target gene’s promoter. For this reason, we incorporate a weight w*_ij_* for the interaction between gene j and CRM i. We chose the simplest linear function to model the dependence of w*_ij_* on the distance d*_ij_* , namely we set w*_ij_* =(d*_max_*-d*_ij_*)/ d*_max_*. In this situation, the weight w*_ij_* ranges between 0 and 1 with the highest value at the promoter and lowest at the distance d*_max_*, which is a free parameter. The value d*_max_* can be interpreted as the maximum distance at which CRMs can activate genes in a given activity class. We assume that CRMs that are further from the promoter than d*_max_* are not influencing the activity of the corresponding gene.

In addition, we utilize insulator protein binding data[9] to help link CRMs to their appropriate target gene. One of the roles of insulator proteins is to block unwanted interactions between enhancers and promoters. Since the enhancer blocking function is typically performed by the binding of a complex of insulator proteins, we require 3 insulator binding proteins (ChIP peaks) to be identified in-between a CRM and a promoter to assume that the interaction between them is blocked. Technically, this is achieved by decreasing the weight w_ij_ of such interaction to 0.

Given the matrices G and A, a method is required to find the optimal value for d_max_. The lower limit for d_max_ is trivially 0. The upper limit is not easy to determine exactly, however we have verified that because of the relative density of insulator ChIP-peaks in the genome, values of d_max_ above 100kbp are not practical as almost all weights for such distances are already 0 because of insulators (Figure S11). Because of the complicated function of the model fitness on the value of d_max_, we use an exhaustive search within the 0-100 kb range to find the optimal value of d_max_

## Integrating the different layers of the model using iterative optimization

So far we described two disjoint parts of the model. However, we can define the likelihood function L, representing the probability of a given model given the observed data (for simplicity we present the formula for a single activity class and omit the index k from all terms),

$$\text{L}=\prod_{\text{j}\in\text{Pos}} \text{G}_{\text{j}}\cdot\prod_{j\in Neg} (1-G_{j})=\prod_{j\in Pos} R_{j}\cdot\left(1-\prod_{i\in\sigma_{j}} (1-A_{i}W_{ij})\right)\cdot\prod_{j\in Neg} (1-R_{j})+R_{j}\cdot\prod_{i\in\sigma_{j}} (1-A_{i}W_{ij})$$

where Pos is the set of genes with positive annotation for k and Neg is the set of genes with negative annotation. The binding data is included in the model by the fact that probabilities of CRM activity Ai are in fact conditional on B*_m_*, and could be replaced in the formulas by the terms P(A*_i_* | B*_i1_*, b*_i2_*…b*_i15_*) .

Given the definition of the likelihood function, it is possible to find the optimal model. As the model contains hidden variables, we need to apply the iterative Expectation Maximization approach[10] to find both the estimates of hidden A*_i_*s as well as the parameters (BNs and d_max_). The iterative procedure is initialized by training the BN on the small set of known enhancers (CAD) and then using this BN to estimate the activity matrix A. Once we have the estimate of the hidden variables, we can proceed with the iteration of alternating steps:

- Maximization (M-step): given the estimates of the hidden values of A, find optimal model parameters BN and d_max_
- Expectation (E-step): given the new parameters BN and d_max_, construct the new estimate of hidden variables A.

This procedure is iterated until the total likelihood of the model stops increasing. EM procedure is guaranteed to improve the model in each step, so the algorithm always converges to a local maximum of the likelihood function.

## Testing a model without known CRM activity data – a simplified 2-layer model

Obtaining experimental data on CRM activity remains a challenge for most organisms. For this reason, our method uses CRM activity data only for initialization of the EM procedure, but does not rely on a large set of CRM activity annotations, making the model more generally applicable. However, in some organisms, the current number of CRMs with known activity may not be enough to even reasonably initialize the BN structure. In order to test, whether this middle layer of the model concerning CRM activity is essential for accurate predictions, we have experimented with a simplified model, consisting only of two layers corresponding to TF binding and gene expression. Instead of using the middle layer with variables corresponding to CRM activity, the model uses Bayesian network to directly link TF binding peaks to gene activity. Consequently, the first layer consists of variables describing the sum of binding events assigned to their respective nearest genes. The Bayesian network is trained on the same dataset of genes with known expression in spatial and temporal classes. We have compared the prediction quality of such a model with the predictions obtained from a 3-layered model trained from the same data. As assessed by the ROC scores of the 2-layered model (see Figure S7) it gives results that are clearly non-random, however significantly less accurate than those of the full model.

## Statistical analysis of Gene-CRM assignment

To assess if by only considering CRM-gene pairs within 100kbps from each other we are making any assumptions that would significantly biases our predictions,, we calculated how many genes are assigned to each CRM and vice-versa as a function of the maximum allowed distance (See Figure S11). This was done in the context of Insulator dependent assignment in comparison to a naïve method not taking Insulators into account. Naturally, if we disregard the positions of insulator elements, we obtain a linear increase of assignment with increasing distance. However, once we take into account the positions of insulators, we can see that little is changing for distances increasing above a certain level. This indicates that limiting our considerations to 100kbps does not significantly influence the results. It is worth pointing out that the number of CRMs assigned to an average gene is similar to the number of genes assigned to an average CRM and is close to 3.

We have also computed the number of cases in which the prediction of a gene’s activity depends on CRMs that are closer to another gene. For genes with known activity, >20% of genes active in one of the tissues of interest, use a CRM that is closer to another gene. Therefore, from the CRMs perspective, it is not regulating the closest proximal TSS. In general, more than 40% of predictions in our model are dependent on a CRM that is closer to another gene.

## List of Supplementary tables

1. List of all used datasets with references

## List of Supplementary datasets

1. Quantitative TF occupancy for 8008 CRMs and 15 different ChIP experiments
2. Distances (<100 000) between transcriptional start sites of 14689 genes and all 8008 CRMs including count of insulator peaks in-between them
3. CRM activity from CAD database in spatial (a) and temporal (b) classes
4. Promoter activity estimates based on histone H3K4 tri-methylation from mod-encode for spatial (a) and temporal (b) expression classes
5. Gene activity, based on in-situ annotations, for spatial (a) and temporal (b) classes – training data
6. New annotations of gene activity based on BDGP release 3 (2010). In spatial (a) and temporal (b) classes
7. Predictions for all genes and all classes made by the full model trained with the EM procedure
8. Manually curated annotations for top 100 VM predictions
